# Modeling of autophagy-related gene expression dynamics during long term fasting in European eel (*Anguilla anguilla*)

**DOI:** 10.1038/s41598-017-18164-6

**Published:** 2017-12-20

**Authors:** Valérie Bolliet, Jacques Labonne, Laure Olazcuaga, Stéphane Panserat, Iban Seiliez

**Affiliations:** 1 INRA, UMR 1224 ECOBIOP, F-64310 St Pée sur, Nivelle, France; 2 Univ Pau & Pays Adour, UMR 1224 ECOBIOP, UFR Sciences et Techniques Côte Basque, Anglet, France; 3 INRA, UMR 1419 Nutrition Metabolisme Aquaculture, F-64310 Saint Pée sur, Nivelle, France; 4 Univ Pau & Pays Adour, UMR 1419 Nutrition Metabolisme Aquaculture, F-40000 Mont de Marsan, France

## Abstract

Autophagy is an evolutionary conserved cellular self-degradation process considered as a major energy mobilizing system in eukaryotes. It has long been considered as a post-translationally regulated event, and the importance of transcriptional regulation of autophagy-related genes (*atg*) for somatic maintenance and homeostasis during long period of stress emerged only recently. In this regard, large changes in *atg* transcription have been documented in several species under diverse types of prolonged catabolic situations. However, the available data primarily concern *atg* mRNA levels at specific times and fail to capture the dynamic relationship between transcript production over time and integrated phenotypes. Here, we present the development of a statistical model describing the dynamics of expression of several *atg* and lysosomal genes in European glass eel (*Anguilla anguilla*) during long-term fasting at two temperatures (9 °C and 12 °C) and make use of this model to infer the effect of transcripts dynamics on an integrated phenotype – here weight loss. Our analysis shows long-term non-random fluctuating *atg* expression dynamics and reveals for the first time a significant contribution of *atg* transcripts production over time to weight loss. The proposed approach thus offers a new perspective on the long-term transcriptional control of autophagy and its physiological role.

## Introduction

Over the last decades, arrivals of European glass eels *Anguilla anguilla* dramatically dropped (by up to 90%), leading to an important decline in continental stocks of eels^1^. In the light of this decline, the European eel was included in 2007 in Appendix II of the Convention on International Trade of Endangered Species (CITES) and was listed in 2008 as critically endangered in the IUCN Red List of Threatened Species. A management framework for the recovery of the European eel stock was established in 2007 by the Council of the European Union. However, the restauration attempts are made difficult by gaps in knowledge of eel biology and the functioning of this panmictic population. Management of the population will benefit of a better understanding of the life cycle of eel and the underlying mechanisms.

The species life cycle spans over a large area and through various ecosystems from the Sargasso Sea, where spawning occurs, to the European continental shelf, where larvae undergo a first metamorphosis into glass eel^2^. At this stage, glass eels display a large panel of migratory tactics, ranging from residency in marine water, to various degrees of upstream colonization through estuarine and freshwater ecosystems^3^. However, these different patterns of migration can have a strong impact on the fate of the population. Indeed, sex determination is environmental: individuals remaining downstream mostly develop in males and return earlier to the Sargasso Sea, whereas individuals colonizing upstream develop mainly into females and stay longer on the continent^4,5^. Therefore, variations in propensity to migrate upstream can have major consequences on the phenotypic structure (sex and size distribution) and thus on the persistence of the species. Such propensity to migrate has been proposed to be related to their energy status. Indeed, most glass eels starve during the estuarine migration^6^ and their ability to reach the upstream zones would closely depend on the mobilization of their energy stores. The propensity to migrate in glass eels may thus results from an adaptation of their metabolism and the establishment of a tightly controlled energy mobilizing system during fasting.

It is now widely accepted that macroautophagy (hereafter referred to as autophagy) plays a major role in mobilizing diverse cellular energy and nutrient stores, including proteins, carbohydrates and lipids during starvation in a large panel of taxa^7^. Autophagy is an evolutionarily conserved process in eukaryotes by which cytoplasmic cargo sequestered inside double-membrane vesicles is delivered to the lysosome for degradation. This ‘self-eating’ process not only rids the cell of intracellular misfolded proteins or damaged organelles but is also a key adaptive response to provide energy when nutrients are scarce. The level of autophagy increases during prolonged periods of cellular stress in many eukaryotic species (from yeast to mammals) in order to conserve energy and promote survival. In this context, and because glass eels undergo strong fasting while colonizing continental ecosystems, genetic- and environment-related variations of autophagy function can strongly affect their migratory ability as well as their sex and their subsequent survival. Therefore, characterization of functional variation of autophagy at an individual level is a key to address the evolutionary adaptive potential of this species in face of environmental changes.

Accumulating evidence indicates that the autophagic response to stress or starvation may proceed in two phases^8^. A rapid increase in the autophagic flux, which occurs within minutes or hours of exposure to stressful conditions and is entirely mediated by post-translational protein modifications, is generally followed by a delayed and protracted autophagic response that relies on the activation of specific transcriptional programs^9^. Such a biphasic mechanism is explained by the fact that under normal circumstances, most cells have relatively high amounts of autophagy-related proteins (ATG) readily available to generate autophagosomes for the first few hours of starvation^10^. But during prolonged starvation, with depletion of available ATG, the demand is accomplished by synthesizing new proteins through transcription and translation to maintain cellular homeostasis^9^.

The mechanisms underlying the regulation of autophagy have long attracted extensive concern of numerous scholars, and its acute transcriptional-independent regulation by nutrient-sensing signaling pathways is now well described. Thus, existing mechanistic knowledge has been used to build mathematical models that predict how the crosstalk between autophagy and other biological processes work together to determine cell fate decision in response to short-term stresses^11–15^. Far less is known about the long-term transcriptional dependent regulation of autophagy. Indeed, although many signaling pathways and transcriptional factors involved in the transcriptional control of autophagy- and lysosome-related genes are now well described^8^, to our knowledge, no data are currently available on the dynamic aspects of this long-term process. Furthermore, while transcriptional regulation of autophagy genes has emerged as an important mechanism for ensuring the somatic maintenance and homeostasis during long period of stress^16^, the relationship between *atg* gene transcription and phenotypic changes remains largely unexplored both at cellular and tissue/organism levels. In our opinion, the key to make progress in this very field is to actually recognize that integrated phenotypes are complex objects that result from various processes which effects accumulate and interact over time. Therefore, our ambition is to target at long-term/temporal dynamics of transcripts to understand the construction of integrated phenotypes – here weight loss.

In the present study, we propose a statistical exploration of the expression of key autophagy- (*ulk1*, *lc3b*, *atg7*, *atg12*) and lysosome- (*cathepsin A*, *cathepsin D*, *cathepsin F*, *cathepsin L*) related genes in glass eel during long-term fasting (60 days). These genes were chosen based on their reported significant role in the biogenesis of autophagosomes^17^ and the protease activity of lysosomes^18–20^, which is recognized to be responsible for the degradation of muscle proteins during fish migration, maturation and starvation^21^. We analyze the general temporal patterns of expression throughout time to detect linear and non-linear trends, and we evaluate inter-individual variation in gene expression. Then, we extend the analysis by testing the hypothesis that autophagy related transcripts produced over time can contribute to changes in an integrated phenotype (the weight loss), and by assessing how these genes interact with markers known to drive energy supply through catabolism of proteins and lipids (i.e., *glutamate dehydrogenase* (*gdh*) and *carnitine palmitoyltransferase I* (*cpt1*), respectively).

In the coming decades, predicted change of rainfall patterns throughout seasons are expected to modify the thermal dynamics in rivers and estuaries^22^. Metabolic demand is increasing with temperature and we hypothesize that autophagy will also increase in glass eels to provide energy for migration. The ability to increase the expression of autophagy- and lysosome-related genes under increasing temperature may thus be critical for migration. In this context, two relatively contrasting temperatures (9 °C and 12 °C) belonging to a natural range are also challenged in the above presented analyses.

## Results

### Gene expression dynamics

Since gene expression measurements require the sacrifice of glass eels, we only had access to punctual gene expression (at the body scale) at the time of death for each individual. Therefore, the statistical approach that follows aimed at using population scale (or group scale) data to infer possible individual dynamics.

We assumed that temporal dynamics of gene expression between individuals of the same species – and the same population by virtue of sampling – share to some extent some similarity when submitted to the same environmental stressors – here, fasting and temperature. Under such hypothesis, since glass eels were sampled at different fasting duration, any non-random pattern in the temporal dynamics of gene expression of the whole population can inform on the temporal dynamics of each individual profile. But non-conversely, it is paramount to realize that a random temporal pattern at the population scale can either indicate fully uncorrelated non-random profiles between individuals, or fully correlated random profiles between individuals, although the latter is far less likely.

We used polynomials of various orders (up to 6 since 7 different sampling dates were available) to model gene expression profiles at population scale, accounting for an effect of experimental temperature. We started with the most complex model as follows:

1 $$\mathrm{log}({E}_{i,j})=\sum _{i,j}({\beta }_{i,j}\times {t}^{i})$$

where *E*_*i*,*j*_ is the predicted gene expression at the *i* ^*th*^ sampling date (starting from 0) and at temperature *j* (considered as a categorical factor). *β*_*i*,*j*_ are the parameters of the gene expression model. We then used a stepwise approach to evaluate the fit of less complex models down to the null model (in which *E*_*i*,*j*_ is a scalar) and we computed adjusted R-squared coefficients to compare the amount of explained variation between the selected models for the different genes.

**Figure 1:**
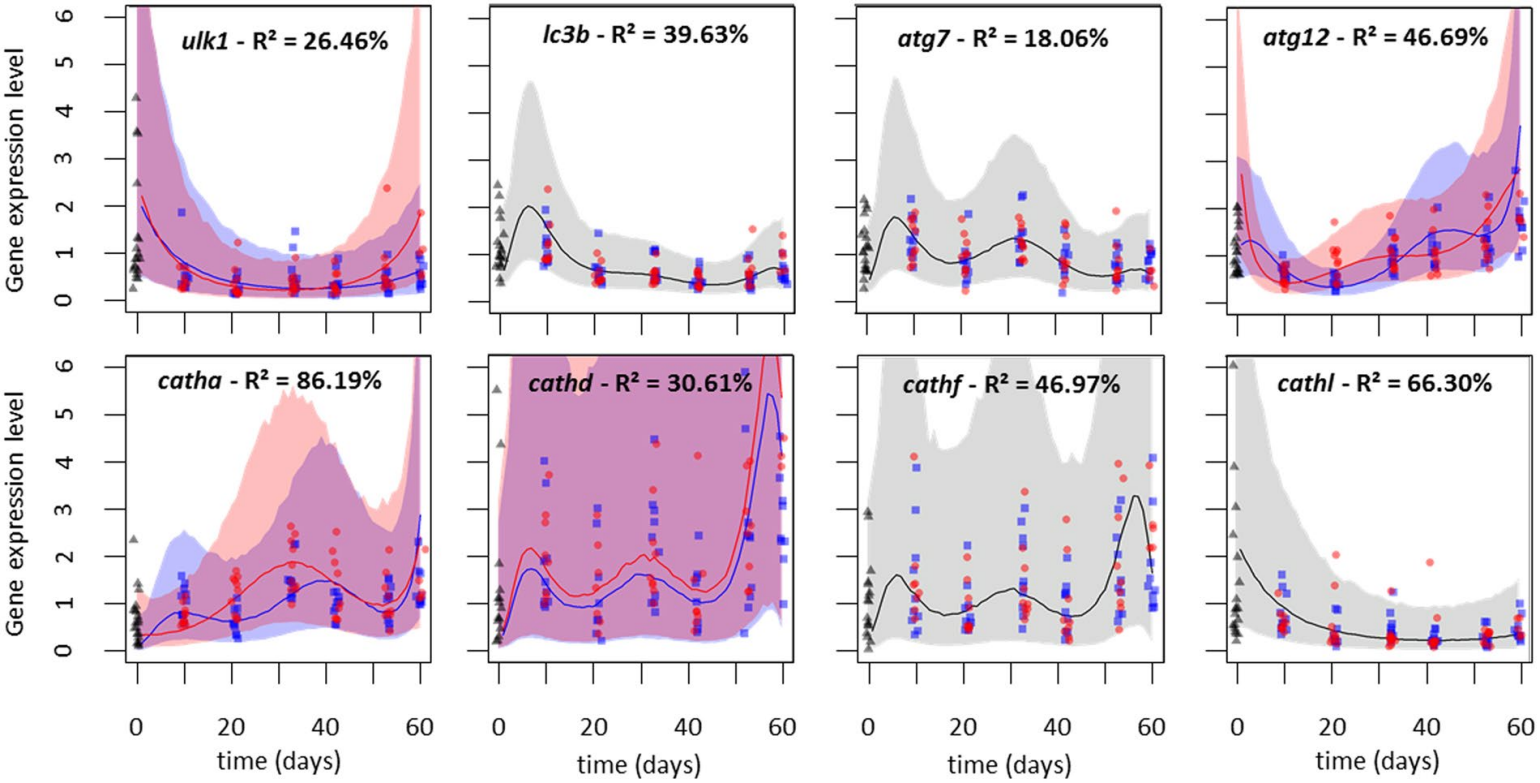
Expression dynamics for autophagy and lysosome related genes. For each gene, blue square symbols indicate data at 9 °C, red circle symbols indicate data at 12 °C, black triangle symbols indicate data at the beginning of the experiment. Artificial jitter was added on the X coordinates to facilitate data perception. Blue and red lines indicate the median of models predictions at 9 °C and 12 °C respectively, the pale blue and pale red surfaces represent the 95% confidence interval of the prediction at 9 °C and 12 °C respectively. When no effect of temperature was detected, the median is indicated by a black line and the surface is displayed in grey. The quality of the model fit for each gene is provided with the adjusted R².

Gene expression dynamics models all exhibited a temporal pattern (Table 1), thereby indicating that none of the considered genes had constant expression through time. Consequently, R² coefficients were all significantly different from 0. Overall, *atg* genes had low R² coefficients (from 18.06% to 46.69%), lysosome-related genes had intermediate to high R² coefficients (30.61% to 86.19%) and metabolism-related genes always had high R² coefficients (73.14% to 87.72%), indicating a stronger inter-individual variation in the former gene groups than in the latter (Fig. 1 and Supplementary online material 1).

**Table 1:** Gene expression models selection.

Genes										
Models	atg7	atg12	lc3b	ulk1	catha	cathd	cathf	cathl	gdh	cpt1
Null model	198.4091	200.9975	195.0637	274.8661	217.5909	303.4851	299.9847	288.2715	211.8984	332.8236
Temperature only model	200.3295	202.651	192.973	271.6297	219.0855	305.1524	301.9706	286.9918	212.971	325.722
Linear model	188.2157	168.6486	162.3492	272.4148	195.9116	297.8339	298.2102	258.8775	211.7331	232.7246
2nd order polynomial	188.0247	142.2302	147.0513	240.335	187.7946	297.0104	296.297	**243**.**8806**	212.6022	216.468
3rd order polynomial	189.7838	130.495	142.1297	239.5692	188.6795	295.394	296.4119	244.4375	202.7846	212.8415
4th order polynomial	191.3504	125.182	143.1294	239.5406	187.5967	297.3427	298.4112	245.4981	199.1094	213.5762
5th order polynomial	185.5638	126.3158	141.1464	241.3106	183.3651	298.9851	300.4111	245.94	199.8602	215.2168
6th order polynomial	**177**.**9332**	126.7975	**135**.**2623**	240.2511	176.1458	295.1183	**294**.**0369**	247.1009	200.1375	213.4855
Linear model + temperature	189.6107	163.6934	164.0462	271.2542	197.3702	297.8925	299.7345	260.7578	213.4121	234.1897
2nd order polynomial + temperature	189.713	139.9847	149.0366	241.0182	188.1298	297.6679	298.145	245.7898	214.0683	218.4512
3rd order polynomial + temperature	191.5685	130.6108	143.9028	240.8709	188.2381	294.633	297.9057	246.4353	202.4471	214.702
4th order polynomial + temperature	193.0919	125.6549	144.8236	241.0375	187.4392	296.628	299.8974	247.4832	198.1059	215.3708
5th order polynomial + temperature	187.4448	126.6208	142.6538	242.7152	182.5773	298.1341	301.8939	247.9396	198.5003	217.049
6th order polynomial + temperature	179.8237	127.0546	136.7759	241.5779	175.0605	**293**.**6412**	295.469	249.1004	198.8159	215.2838
Linear model * temperature	191.4915	152.4921	165.7432	271.1041	195.8631	299.7542	301.6908	261.5845	214.1236	218.4733
2nd order polynomial * temperature	191.8085	140.6064	144.8647	**238**.**9733**	186.3509	300.0211	301.5847	249.0721	216.6558	**208**.**6308**
3rd order polynomial * temperature	195.7395	133.0582	141.3383	242.2301	189.1963	298.8941	301.3621	251.8635	205.4194	211.4949
4th order polynomial * temperature	191.7946	112.8464	140.6139	241.6344	173.383	301.2723	304.9136	250.3146	**197**.**6711**	212.7588
5th order polynomial * temperature	188.5724	**112**.**5853**	138.6162	243.1846	**170**.**3905**	300.6816	304.1199	252.6209	198.3532	214.3907
6th order polynomial * temperature	187.5341	114.5738	137.9766	244.8093	171.4527	301.4109	302.7866	254.381	200.1839	214.5539

For each gene, the Akaike Information Criterion (AIC) has been calculated; the minimal value is underlined, indicating the best trade-off between parsimony and fit. The null model represents the mean of data, the simplest possible model.

Temporal dynamics were always non-linear, with at least a quadratic term (for *ulk1*, *cathl* and *cpt1*) to represent this non-linearity (Table 1, Fig. 1). All other monitored genes displayed a more fluctuating expression pattern with an upward trend for *atg12* and lysosomal genes (*catha*, *cathd* and *cathf*) and a downward trend for *lc3b and atg7* (Fig. 1).

Finally, the temperature had no effect on some genes expression *(atg7*, *lc3b*, *cathf* and *cathl*) and interacted with the temporal effect for other genes expression (*atg12*, *ulk1*, *catha*, *gdh* and *cpt1*) (Table 1).

### Inference of transcripts dynamics effects on weight loss

We hypothesized that gene transcripts quantities can be a limiting factor for cellular functions such as autophagy, especially for long term sustainability^9,16,23–25^. The usual approach, however, is to estimate simple correlations between punctual levels of gene expression, and the phenotype (here weight loss). We advocate that such approach is unlikely to underpin real relationships between transcripts dynamics and integrated phenotypes, since weight loss is calculated over time (days or weeks of experiment in our case). We therefore aimed at calculating the cumulated production of transcripts over time, based on the gene expression dynamics models previously described and infer the effect of the mean of this quantity on weight loss (shown in Supplementary online material 2).

However, because weight loss is a cumulative data, it is important to notice that any random gene expression profile will be likely to explain a large part of the observed variation in weight loss, especially if i) weight loss is linear over time and ii) the variance of the random gene expression is reduced. The approach used here is to rely on non-linear variation in weight loss, as well as on non-random gene expression profiles. To efficiently assess the effect of gene expression profiles on weight loss, we therefore generated a null gene expression model depicting the variation of a randomly expressed gene (*E*_*null*_), with a variance being the average of observed variances in our dataset. The role of this null gene expression model is to correlate to the linear increase of weight loss, thereby avoiding to mistakenly attribute any linear variation to other genes of interest. The cumulated production of transcripts for the randomly expressed gene at time *i* and temperature *j* was expressed as:

2 $$P(null,i,j)={\int }_{0}^{i}{E}_{nul{l}_{i,j}}\,{d}_{i}$$

We then restrained our exploratory analysis to 6 genes of interest: first, we considered 4 mandatory autophagy related genes (*ulk1*, *lc3b*, *atg7*, *atg12*). If one of these transcripts is lacking in the cell, then the whole construction of the autophagosome is halted. To account for this dependence, we therefore calculated the cumulated production of transcripts for autophagy *P(A)* at time *i* and temperature *j* as the integral of the product of autophagy related transcripts in the cell as follows:

3 $$P(A,i,\,j)={\int }_{0}^{i}{E}_{ulk{1}_{i,j}}\times {E}_{lc3{b}_{i,j}}\times {E}_{atg{7}_{i,j}}\times {E}_{atg{12}_{i,j}}\,{d}_{i}$$

The calculation of cumulated production of transcripts for *atg* genes (*P(A)*, Supplementary online material 3A) showed a nonlinear accumulation of this production: production was high during the first ten days, then stabilized at a slower rate before raising again during the last ten days. This pattern was more pronounced at high temperature.

Then, we also considered key actors of the catabolism of amino acids and lipids respectively. We calculated the cumulated production of transcripts for *gdh P(B)* and for *cpt1 P(C)* at time *i* and temperature *j* as follows:

4 $$P(B,i,j)={\int }_{0}^{i}{E}_{gd{h}_{i,j}}\,{d}_{i}$$

5 $$P(C,i,j)={\int }_{0}^{i}{E}_{cpt{1}_{i,j}}\,{d}_{i}$$

For *gdh*, the cumulated production of transcripts (*P(B)*, Supplementary online material 3B) followed a near linear pattern at low temperature, whereas it became nonlinear at high temperature: production was initially higher up to the 20^th^ day, and then paced down to a lower rate. Regarding *cpt1*, the cumulated production of transcripts (*P(C)*, Supplementary online material 3C) displayed a logarithmic pattern, constantly slowing over the duration of the experiment. *P(C)* was higher at high temperature.

Then, we assumed that *gdh* and *cpt1* expression could be linked either to autophagy related catabolism, or to autophagy independent catabolism. Indeed, under fasting conditions, protein catabolism (both autophagy-dependent and non-dependent) results in the production of free amino acids, which are used by several enzymes including GDH to fuel the tricarboxylic acid (TCA) cycle for energy production^26^. Similarly, fatty acids produced by lipophagy as well as cytosolic lipases are transported into mitochondria through the carnitine palmitoyltransferase system (including the outer mitochondrial membrane protein CPT1) and enter the fatty acid β-oxidation pathway to produce acetyl-CoA, which fuels the TCA cycle and energy production^7^. The flux of fatty acid β-oxidation pathway is primarily determined by CPT1^27,28^. The general inference of the cumulated production of transcripts on weight loss (*W*) at time *i* and temperature *j* was thus modelled as:

6 $$\begin{array}{c}{W}_{i,j}={\gamma }_{j}\times [{\alpha }_{1,j}\times P(A,i,\,j)+{\alpha }_{2,j}\times P(A,i,\,j)\times P(B,i,\,j)+{\alpha }_{3,j}\times P(A,i,\,j)\\ \,\,\,\,\,\,\,\,\,\,\,\times P(C,i,\,j)+{\alpha }_{4,j}\times P(B,i,\,j)+{\alpha }_{5,j}\times P(C,i,\,j)+{\alpha }_{6,j}\times P(null,i,\,j)]\end{array}$$

where *γ*_*j*_ is a temperature dependent scaling parameter for the general effect of transcripts on weight loss *W*_*i*,*j*_, and *α*_*1*,*j*_, *α*_*2*,*j*_, *α*_*3*,*j*_, *α*_*4*,*j*_, *α*_*5*,*j*_ and *α*_*6*,*j*_ are the relative contributions (i.e., summing to one) of the different transcripts and their interactions sampled in a Dirichlet distribution. *P(C)* was relatively lower at 12 °C than at 9 °C (Supplementary online material 3A).

We compared the above presented model (termed as M1) where *P(A)*, *P(B)*, *P(C)* and *P(null)* are included, to a model where only the effect of *P(null)* was inferred on weight loss (M0). When comparing the M0 inference model (where only the random gene expression was used) and the M1 model (where *P(A)*, *P(B)* and *P(C)* and their interactions were also used), the DIC value indicated the M1 model as the best compromise between fit and complexity to explain weight loss data (DIC_M1_ = 921.1, DIC_M0_ = 926.4). In the M1 model, the relative contributions of *atg* genes, *gdh* and *cpt1* on weight loss differed as a function of temperature, although a general pattern emerged. The linear trend explained by randomly expressed gene (*E*_*null*_) represented 30.64% of the contribution at 9 °C versus 51.71% at 12 °C. The general effect of transcript production on weight loss was higher at 12 °C (*γ* *=* 1.104, SD = 0.147) than at 9 °C (*γ* *=* 0.8738, SD = 0.2038). At 9 °C, the biggest contributor to weight loss was *P(B)* (*gdh*), followed by *P(C)* (*cpt1*) both independently of *P(A)*. The contribution of *atg* genes *P(A)* ranked third, closely followed by the interactive effect of *P(A)* and *P(B)*. The interactive effect of *P(A)* and *P(C)* ranked last (Table 2). At 12 °C, the relative contribution of *P(B)*, *P(A)* and their interaction increased, whereas the contribution of *P(C)*, and its interaction with *P(A)* decreased.

**Table 2:** Relative contributions (reported in %) of *atg* genes, *gdh* and *cpt1* and their interaction in the inference model for weight loss.

Process	Relative contributions^a^	
	9 °C	12 °C
*atg* genes	15.31% (SD = 0.137)	17.84% (SD = 0.149)
*atg* genes * *gdh*	14.91% (SD = 0.134)	15.74% (SD = 0.138)
*gdh*	39.33% (SD = 0.19)	44.6% (SD = 0.205)
*atg* genes * *cpt1*	11.37% (SD = 0.11)	6.57% (SD = 0.069)
*cpt1*	19.09% (SD = 0.159)	15.25% (SD = 0.135)

^a^The contributions have been scaled to 100% after removing the linear trend, that explained 30.64% and 51.71% of the weight loss at 9 °C and 12 °C respectively.

## Discussion

Monitoring the dynamics of biological processes as well as the impact of these dynamics on the establishment of integrated phenotypes is of paramount importance for a better understanding of both the processes themselves and their role at the whole organism-level. In that aim, we conducted a novel approach to (i) build a statistical model for the dynamics of expression of several *atg* and lysosomal genes in glass eel during long-term fasting at two temperatures (9 °C and 12 °C) and (ii) make use of this dynamic model to test whether or not, and to what extent, the autophagy related transcripts dynamics can contribute to changes in an integrated phenotype – here weight loss.

Our analysis predicts that most of monitored autophagy- and lysosome-related genes display a non-random fluctuating expression pattern during long-term fasting in glass eels. Such a long-term temporal pattern of gene expression dynamics is consistent with the currently accepted idea that gene transcription events control long-term autophagy flux^9,16,23–25^. Indeed, the adaptive autophagic stress response has been referred to as being biphasic. Thus, after a rapid autophagic response, which involves the post-translational modification of cytosolic proteins, a collection of transcription factors upregulates the expression of genes encoding proteins that sustain and regulate autophagic flux. In this regard, the predicted fluctuating expression pattern of autophagy- and lysosomal-related genes in glass eel during long-term fasting could indeed capture some of the successive waves of replenishment of critical proteins of this function.

However, besides the fluctuating pattern, our model also predicts upward or downward expression trends according to the considered genes. It is thus interesting to note that *lc3b*, *atg7* and *cathl* (as well as *ulk1* for the first 40 days of fasting) showed a rather high expression level at the beginning followed by a constant decline, possibly reflecting a fall in autophagic capacity in long-term fasting glass eels. The result obtained for *lc3b* particularly caught our attention. Indeed, previous studies from both yeast and mammalian models supported a positive correlation between the concentration of LC3b and the mean autophagic vesicle size^29,30^. Notably, mutant yeast strains engineered for reduced *atg8* (*lC3b* homolog in yeast) expression accumulate autophagosomes smaller than wild-type yeast in response to starvation^30^. Within the mutant strains, the more substantial the *atg8* reduction, the smaller the size of resulting autophagosomes^30^. More recently, Martin *et al*. developed a computational model describing autophagic vesicle dynamics in a mammalian system and also reported a positive correlation between LC3 levels and autophagic vesicle size across single cells, in line with a proposed function for LC3 in phagophore elongation^14^. Collectively, these studies in combination with the temporal downward expression trend of *lc3b* predicted by our model support a decrease in autophagic vesicle size, and therefore in the autophagic capacity, in glass eels during prolonged starvation. These results are consistent with earlier studies showing that protein breakdown through macroautophagy gradually decline in organs such a liver during long-term fasting but remains sustained through Chaperone-Mediated Autophagy (CMA), a selective form of autophagy that does not require the involvement of any of the *atg* genes monitored in the present study^31^. However, it remains to be established whether or not CMA or a CMA-like process exists in fish. In that condition, the predicted upward trend in the expression of some other studied genes (*atg12*, *catha*, *cathd*, *cathf* and also *ulk1* at the end of the experiment) may appear contradictory. However, we and other have previously demonstrated that induction of *atg* and lysosomal transcripts can also occur as a compensatory response to autophagy inhibition^32–35^. The underlying mechanisms are now well described and would at least partially involve the transcription factor TFEB recognized as a major player in the regulation of the expression of *atg* and lysosomal genes^36^. In any case, our analysis highlights the complexity of the regulatory network involved in the autophagy-lysosomal system.

Such a complexity is further revealed when considering the R² coefficient that compares the amount of explained variation between the selected models for the different genes. Indeed, the lower R² coefficients for *atg* genes (from 18.06% to 46.69%) than for lysosomal genes (30.61% to 86.19%) could be explained by the existence of a multiplicity of selective autophagy that converges to a common lysosomal degradative system. Indeed, autophagy was long considered to be a nonselective degradation pathway^37–41^. However, recent research has revealed that autophagy is also critical for the selective degradation of specific cargos, such as aggregated proteins (aggrephagy), endoplasmic reticulum (ER-phagy or Reticulophagy), mitochondria (mitophagy), peroxisomes (pexophagy), lipid droplets (Lipophagy), glycogen (Glycophagy) and even invading pathogens (xenophagy)^42–45^. All these types of selective autophagy require the formation of vesicles and therefore the transcription of the *atg* genes monitored in the present study. However, emerging evidence suggests that the size as well as the number of the autophagosomes not only regulate efficiency, but may also be critical for the cargo selectivity^46^. The relative strong inter-individual variation observed for the monitored *atg* genes may therefore reflect this multiplicity of autophagy-related mechanisms likely activated during fasting.

Finally, contrary to what we might have thought, we do not observe a general trend of the effect of temperature on *atg* and lysosomal gene expression dynamics. The observed effects are relatively slight and concern only a few genes (*ulk1*, *atg12*, *catha* and *cathd*) at certain sampling times. Only one gene (*cathd*) exhibit a slightly higher expression level at 12 °C than at 9 °C throughout the fasting period. However, these data do not indicate that the dynamics of expression of these genes cannot be affected by temperature, but rather that at the tested temperatures (which correspond to a natural range) few if no effect is observed. Could this apparent low ability of eels to increase the expression of autophagy- and lysosome-related genes under increasing temperature be critical for their migration in the future?

Now, what are the use of these transcripts dynamics when looking at integrated phenotype? We know that autophagy (in its narrow definition of this paper) is only a part of the catabolic chain that provides energy during a fasting period. We therefore wanted to weigh the contribution of the autophagy related transcripts produced over time on an integrated phenotype (here the weight loss) by considering how these genes interact with markers known to drive energy supply through catabolism of proteins and lipids. Looking at the accumulation of *atg* genes, *gdh*, and *cpt1*, it was clear that the dynamics of these 3 groups of transcripts strongly differed during our experiment, which allowed for further inference on weight variation. We found the contribution of autophagy related transcripts dynamics to be significant and quite stable when temperature changed (between 41.58% at 9 °C and 40.15% at 12 °C, summing all terms where autophagy is involved in Table 2). This relatively stable contribution of *atg* genes between the two temperatures may be linked to the wide range of different processes mentioned above (lipophagy, glycophagy, mitophagy, pexophagy etc…) that all depend on the studied genes and that may have been affected differently by temperature. By comparison, the contribution of *cpt1* dramatically reduced at higher temperature, compensated by a relative increase of *gdh* contribution, in line with a preferred use of amino acids relative to fatty acids for energy supply during starvation in European eel^47^. To our knowledge, this is the first demonstration of the effect of *atg* transcripts dynamics on an integrated phenotype. In recent years, a major effort has been made to understand the mechanisms underlying the regulation of *atg* gene expression as well as the role of these nuclear events in the control of cellular homeostasis during long period of stress^9,16,23–25^. However, usual approaches using simple correlations between punctual levels of gene expression and integrated phenotypes failed to capture such functional and dynamic relationships between transcripts and integrated phenotypes.

Overall, the model presented here is consistent with published findings and improved, to some extent, our understanding of weight loss dynamics under long-term fasting and in different temperatures. However, it is currently limited to only minimal molecular details (i.e., 4 transcripts that are mandatory for autophagy). In the future, an extended model, based on the framework presented here and including additional known molecular factors involved in the different types of selective autophagy will certainly provide a better description of temporal individual variation in the integrated phenotype. Additionally, better estimates and results will be obtained using biological models where multiple sampling over time for gene transcription is an option, thereby relaxing our assumption of similarity in the pattern of expression dynamics between individuals, thus simplifying the first stage of our approach.

### Wrapping-up

Despite these shortcomings, our results hint at the role of *atg* transcripts as potential indicators of ability to cope with long-term fasting in young eels. Linking these results with the sources of variation in gene transcripts dynamics therefore opens exciting research paths to understand (i) finer regulations mechanisms of autophagic response to long-term fasting, (ii) sources of inter-individual variation in ability to cope with environmental variation of resources during migration, a source of growing concern in the various ecosystems occupied by European eels^48^. The adaptive potential of the species is indeed at stake here, since it is also known that determinism of sex is environmentally controlled, especially by growth rate and population densities: on the one hand, the inability of some individuals to sustain long term fasting under changing conditions could affect density distribution along the various ecosystems that stem the European eel life cycle. Yet on the other hand, the control of autophagy on weight loss will interactively affect sex determinism and thereby life cycle length and migration paths. We therefore advocate for further research to focus on long term analysis of gene expression and relationship to phenotypic variation. Based on the present results, we deem it very unlikely that non-random temporal dynamics of gene transcripts, when detected, may contain no information on the dynamics of integrated phenotypes.

## Materials and Methods

### Origin and handling of fish

Glass eels were caught in March 2014 (authorization of scientific fishing, 17 September 2013) on the Atlantic coast, downstream from the mouth of the Huchet river (Moliets, South-Western France, 43°51N, 1°23W). Fishing took place by night during flood tide using a hand net. After collection, glass eels were brought to the laboratory and kept in a tank at 11 °C. The following morning, 150 glass eels were anaesthetized by immersion in a solution of benzocaine (0.3 mL L^−1^), measured (±0.5 mm), weighed (Sartorius CP 153 balance, ±1 mg) and individually tagged using visible implant elastomers as described previously^49^.

### Experimental design

Ten Aquaria (50 cm long, 25 cm large and 25 cm high, 20 liters) filled up with tap water to 15 cm high (outflow height) were placed in two thermoregulated rooms in order to get five aquaria at 9 °C and the five others at 12 °C. Water was re-circulated through a filter and UV light. Four pieces of artificial vegetation mimicking *Elodae* shelters (10 cm high) were placed on the floor of each aquarium, regularly spaced along a line at 10 cm from the front glass of the aquarium. Photoperiod was 12 L/12D with dim light during the light phase (27 μW/cm^2^ ≈ 4 lux) and 30 minutes of dawn and dusk. Temperature was measured every day and the water level adjusted every week with aerated tap water kept at room temperature.

Fifteen tagged glass eels were transferred in each aquaria (Fourteen for analyses and one additional in case of mortality). There was no initial significant difference between glass eels placed at 9 °C versus 12 °C in weight (Welch 2-samples t-test, t = 1.5198, 131 df, p = 0.131) or length (t-test, t = 0.51773, 131 df, p = 0.6055). Two individuals were randomly sampled in each tank every 10 days during a period of 60 days. Glass eels were immediately anaesthetized, weighted and lengthened as described above, frozen in liquid nitrogen and then kept at −80 °C until analysis. No mortality was observed and the remaining fish were not used for analyses.

Procedures used in this study have been validated by the ethics committee ‘Aquitaine poissons-oiseaux’, the 15 January 2013. The experiment was carried out in strict accordance with the EU legal frameworks, specifically those relating to the protection of animals used for scientific purposes (i.e., Directive 2010/63/EU), and under the French legislation governing the ethical treatment of animals (Decret no. 2013-118, February 1st, 2013). The INRA/UPPA experimental station is certified for animal services under the permit number B64-495-2 by the French veterinary services, which is the competent authority.

### Behavioral observations

Fish behavior was observed every morning during the 60 days of experiment. Observations took place at 70 cm away from the front of the aquaria avoiding any visual disturbance. Each aquarium was observed during 5 minutes: Glass eels were identified by their tagging and individual activity (swim or rest) and location (floor, shelter or water column) were noted. At 9 °C, all glass eels remained inactive, hidden in the shelters or on the bottom while at 12 °C, eight glass eels were observed in more of 70% of the observations swimming in the water column or along the wall of the aquarium. As autophagy is related to fasting but also to locomotor activity and stress, these eight glass eels were removed from our analysis.

### mRNA levels analysis: quantitative RT-PCR

The extraction of total RNA was performed using TRIzol reagent (Invitrogen, 15596018) according to the manufacturer’s recommendations. One microgram of the resulting total RNA was reverse transcribed into cDNA, using the SuperScript III RNAseH-reverse transcriptase kit (Invitrogen, 18080085) with random primers (Promega, Charbonniéres, France) according to the manufacturer’s instructions. Primers specific to autophagy- (*ulk1*, *lc3b*, *atg7*, *atg12*) and lysosome- (*cathepsin A*, *cathepsin D*, *cathepsin F*, *cathepsin L*) related genes as well as specific to *glutamate dehydrogenase* (*gdh*) and *carnitine palmitoyltransferase I* (*cpt1*) were designed using Primer3 software (version 4.1.0)^50,51^. To confirm specificity of the newly developed RT-PCR assay, the amplicon was purified and sequenced (Beckman-Coulter Genomics, Takeley, UK). The primers used for RT-PCR assays are listed in Table 3. Quantitative RT-PCR was carried out on the Roche LightCycler 480 System (Roche Diagnostics, Neuilly sur Seine, France). The assays were performed using a reaction mix of 6 µl per sample, each of which contained 2 µl of diluted cDNA template, 0.24 µl of each primer (10 µM), 3 µl Light Cycler 480 SY Green Master mix (Roche Diagnostics, 4887352001) and 0.52 µl DNAse/RNAse-free water (5 Prime GmbH, 2500020). The PCR protocol was initiated at 95 °C for 10 min for initial denaturation of the cDNA and hot-start Taq-polymerase activation, followed by 45 cycles of a 3-step amplification program (15 s at 95 °C; 10 s at 60 to 64 °C and 15 s at 72 °C), according to the primer set used. Melting curves were systematically monitored (temperature gradient at 1.1 °C/10 s from 65 to 94 °C) at the end of the last amplification cycle to confirm the specificity of the amplification reaction. Each PCR assay included replicate samples (duplicate of reverse transcription and PCR amplification, respectively) and negative controls (reverse transcriptase- and cDNA template-free samples, respectively). For the expression analysis of mRNA, relative quantification of target gene expression was performed using the delta CT method described by Pfaffl^52^. The relative gene expression of eukaryotic translation elongation factor 1 alpha 1 (Eef1a1) was used for the normalization of measured mRNA. Its expression showed an acceptable coefficient of variation (CV = 0.296), a positive linear temporal trend was detected but it was too small compared to the temporal variation in expression of other genes to affect our conclusions (Supplementary online material 4). In all cases, PCR efficiency (E) was measured by the slope of a standard curve using serial dilutions of cDNA (1/20, 1/40, 1/80, 1/160, 1/320). In all cases, PCR efficiency values ranged between 1.8 and 2.2.

**Table 3:** Sequences of the primer pairs used for real-time quantitative RT-PCR.

Gene	5′/3′ Forward primer	5′/3′ Reverse primer
**Autophagy-related genes**		
*ulk1*	GACTTTAACGGAGGCGAGTG	CCAGTCGTGTTTCTCCTTATGTC
*lc3b*	TACAGGACATAGGCCGCTAA	ACTCGCTGTTCAAATGTCCT
*atg7*	CGGCTGAGATCTGGGACA	AGCCAGATTGAGCGACTGAT
*atg12*	GCGGTAGGGGACACTCCTAT	CACTGCCAAAAACATTCAAATAAC
**Lysosome-related genes**		
*catha*	GGGAACAAGCACCTGCATTA	CGCCATCATCCTGAATTAGA
*cathd*	GGACATCCCATGCTCTTGTT	CCGTAATACTGAGCATCAAGGT
*cathf*	GGGATATGGACATCGTAATGG	GCAGATGGGCGTTGTTTAAT
*cathl*	TCAGTTCTACCAATCTGGAATCTAC	CTTCCTGTCTTTGGCCATGT
**Metabolism-related genes**		
*gdh*	TACGCTCAGAACATGGTTGC	AAGCCGTAGGGGAAAAAGAA
*cpt1*	ATTTGGACCGGTGGCTGAT	CCTCGAATCACATCTTGTTTTCC
**Reference gene**		
*eef1a1*	CACCGAGACGGAACCTTAAA	GACCCCTTCCCCATCTCA

### Data modelling

We used R Software (R 3.2.2)^53^ to fit gene expression polynomials and determinate optimal orders through Akaike Information Criterion (AIC) stepwise selection approach, so to obtain a balance between model fit quality, and complexity of the model (number of parameters). We then modelled gene expression profiles (as deduced from the previous selection) and their effect on weight loss in a single Hierarchical Bayesian model (See Supplementary online material 5), using non informative Gaussian priors for all gene expression models parameters. We used a [0;1000] uniform distribution prior for the *γ*_*i*_ parameter, and equiprobable priors (1;1;1;1;1;1) for the Dirichlet distribution. The joint posterior distribution of all hyper parameters of the model was approximated by MCMC sampling as implemented by the OpenBUGS (version 3.2.3) software. A MCMC sample of 10000 draws was used, after checking its convergence by applying the Gelman-Rubin test^54^. The fit of models to observed weight loss data was assessed using the Deviance Information Criterion (DIC)^55^, that balances the quality of the fit (maximum likelihood) by the complexity of the model (number of estimated parameters). Such an approach is best suited when one desires to account for parsimony as much as for predictive power.

### Data availability

All data generated or analyzed during this study are included in this published article (and its Supplementary Information files).

## Electronic supplementary material

Supplementary material
