# Supplementary material for: Modeling of autophagy-related gene expression dynamics during long term fasting in European eel (Anguilla anguilla)
Source: Sci Rep. 2017 Dec 20;7:17896. doi: 10.1038/s41598-017-18164-6 (PMC5738402; doi:10.1038/s41598-017-18164-6)
Supplement: Supplementary file 1 — Supplementary material [file 41598_2017_18164_MOESM1_ESM.pdf]

Supplemental Information

Modeling of autophagy-related gene expression dynamics during long term fasting in European eel (*Anguilla anguilla*)

Valérie Bolliet, Jacques Labonne, Laure Olazcuaga, Stéphane Panserat, Iban Seiliez

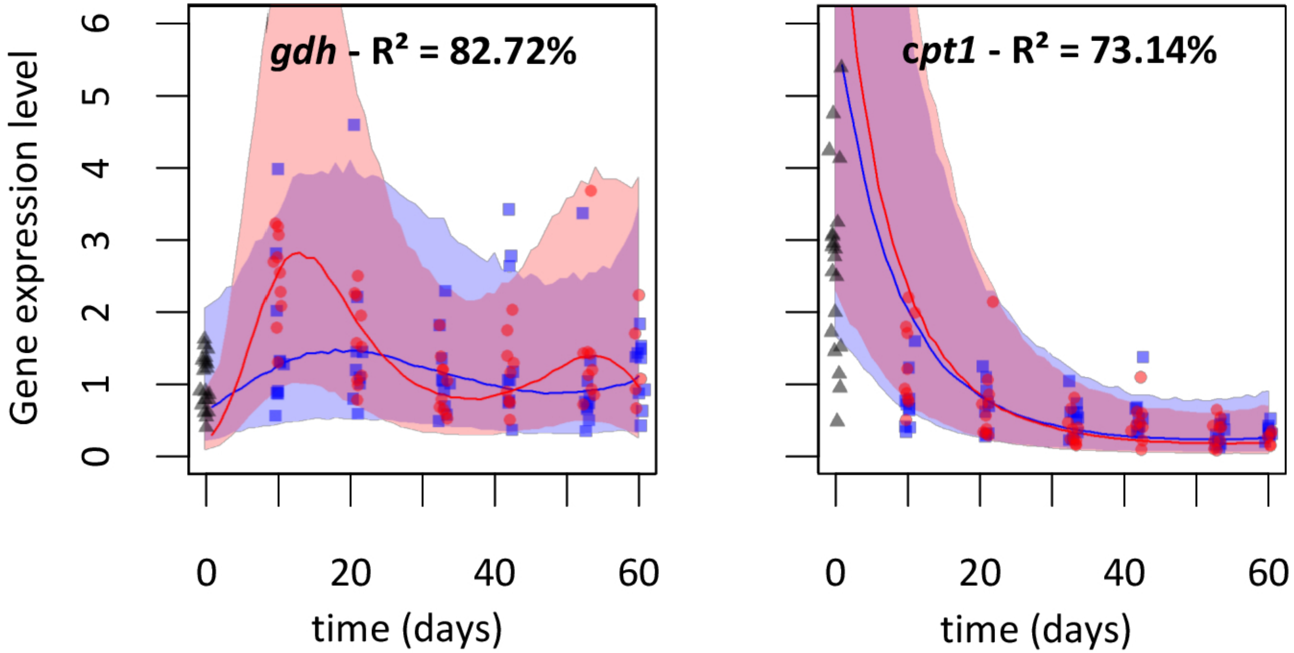

Supplementary online material 1: Expression dynamics for catabolism related genes. For each gene, blue square symbols indicate data at 9°C, red circle symbols indicate data at 12°C, black triangle symbols indicate data at the beginning of the experiment. Blue and red lines indicate the median of models predictions at 9°C and 12°C respectively, the pale blue and pale red surfaces represent the 95% confidence interval of the prediction at 9°C and 12°C respectively. The quality of the model fit for each gene is provided with the adjusted R^2.

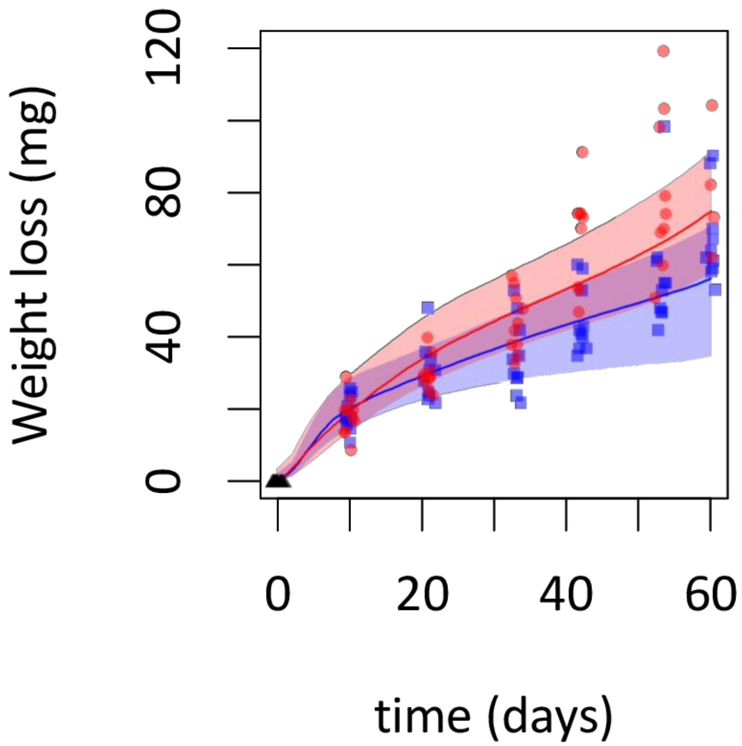

Supplementary online material 2: Weight loss in mg over time. Blue square symbols indicate data at 9° C, red circle symbols indicate data at 12° C, black triangle symbols indicate data at the beginning of the experiment. Artificial jitter was added on the X coordinates to facilitate data perception. Blue and interrupted lines indicate the median of models predictions at 9° C and 12° C respectively, the pale blue and pale red surfaces represent the 95% confidence interval of the prediction at 9° C and 12° C respectively.

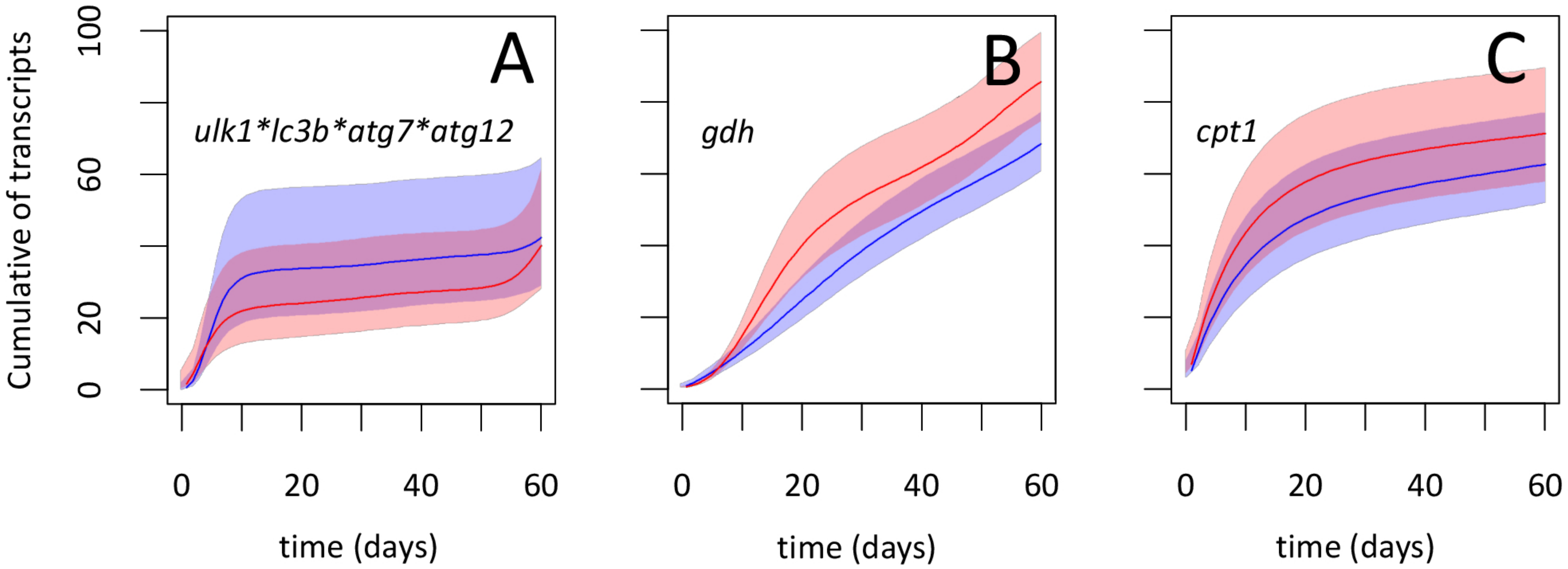

Supplementary online material 3. Cumulative of transcripts production over time for A) autophagy related genes P(A), B) gdh P(B) and C) cpt1 P(C). The blue and red lines indicate the median predictions from the model at 9°C and 12°C respectively, the pale blue and pale red surfaces represent the 95% confidence interval of the prediction at 9°C and 12°C respectively.

Supplementary Online Material 4 : Variation of Eefla1 expression

The coefficient of variation for Eefla1 was 0.296.

The fit of a linear model using time (in days) as a covariate indicated a positive trend, suggesting that Eefla1 expression was 33% higher for samples at day 60 compared to samples at day 0. The percentage of variation explained was low (R^2=0.09665).

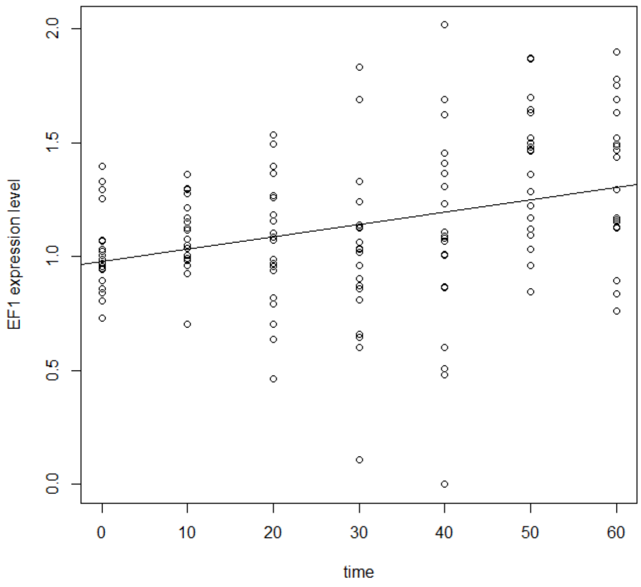

Figure: Eefla1 expression level as a function of sampling time. The full line indicates the linear best fit.

Analysis results (R software 3.4.0)

lm(formula = Eefla1M ~ time)

Residuals:
```
    Min       1Q   Median       3Q      Max
-1.19615 -0.15219 -0.00971  0.21518  0.82183
```

Coefficients:
```
            Estimate Std. Error t value Pr(>|t|)

(Intercept) 0.979796   0.048960  20.012  < 2e-16 ***
time        0.005410   0.001358   3.984 0.000109 ***
---
Signif. codes:  0 '***' 0.001 '**' 0.01 '*' 0.05 '.' 0.1 ' ' 1
```

Residual standard error: 0.3213 on 138 degrees of freedom
Multiple R-squared: 0.1032,   Adjusted R-squared: 0.09665
F-statistic: 15.87 on 1 and 138 DF, p-value: 0.0001092

Supplementary online material 5: Openbugs code, data, and Markov chain initial values for the gene expression and inference model.

```
#################
### MODEL ######
################


model {
#likelhihood
#building gene expression models, effect of temperature.
	for (i in 1:N) {
		ulk1[i] ~dlnorm(ma[i],taua)
		ulk1more[i] ~dlnorm(ma[i],taua)		# sampling to build model fit
		ma[i] <- a[1] + a[2]*temp[i] + a[3]*time1[i]+a[4]*time2[i]+a[5]*time1[i]*temp[i]
+a[6]*time2[i]*temp[i]

		atg12[i]~dlnorm(mb[i],taub)
		atg12more[i]~dlnorm(mb[i],taub)		# sampling to build model fit
		mb[i] <- b[1] + b[2]*temp[i] +
b[3]*time1[i]+b[4]*time2[i]+b[5]*time3[i]+b[6]*time4[i]+b[7]*time5[i] +
	b[8]*time1[i]*temp[i]+b[9]*time2[i]*temp[i]+b[10]*time3[i]*temp[i]+b[11]*time4[i]*temp[i]+b[12]*ti
me5[i]*temp[i]

		lc3b[i]~dlnorm(mtc[i],tauc)
		lc3bmore[i]~dlnorm(mtc[i],tauc)		# sampling to build model fit
		mtc[i]<- c[1]+
c[2]*time1[i]+c[3]*time2[i]+c[4]*time3[i]+c[5]*time4[i]+c[6]*time5[i]+c[7]*time6[i]

		atg7[i]~dlnorm(md[i],taud)
		atg7more[i]~dlnorm(md[i],taud)		# sampling to build model fit
		md[i]<-
d[1]+d[2]*time1[i]+d[3]*time2[i]+d[4]*time3[i]+d[5]*time4[i]+d[6]*time5[i]+d[7]*time6[i]

		catha[i]~dlnorm(me[i],taue)
		cathamore[i]~dlnorm(me[i],taue)		# sampling to build model fit
		me[i] <- e[1] + e[2]*temp[i] +
e[3]*time1[i]+e[4]*time2[i]+e[5]*time3[i]+e[6]*time4[i]+e[7]*time5[i] +
	e[8]*time1[i]*temp[i]+e[9]*time2[i]*temp[i]+e[10]*time3[i]*temp[i]+e[11]*time4[i]*temp[i]+e[12]*ti
me5[i]*temp[i]

		cathd[i]~dlnorm(mf[i],tauf)
		cathdmore[i]~dlnorm(mf[i],tauf)		# sampling to build model fit
		mf[i] <- f[1] + f[2]*temp[i] +
f[3]*time1[i]+f[4]*time2[i]+f[5]*time3[i]+f[6]*time4[i]+f[7]*time5[i] + f[8]*time6[i]

		cathf[i]~dlnorm(mg[i],taug)
		cathfmore[i]~dlnorm(mg[i],taug)		# sampling to build model fit
		mg[i] <- g[1] + g[2]*time1[i]+g[3]*time2[i]+g[4]*time3[i]+g[5]*time4[i]+g[6]*time5[i] +
g[7]*time6[i]

		cathl[i]~dlnorm(mh[i],tauh)
		cathlmore[i]~dlnorm(mh[i],tauh)		# sampling to build model fit
		mh[i] <- h[1] + h[2]*time1[i]+h[3]*time2[i]

		gdh[i]~dlnorm(mm[i],taum)
		gdhmore[i]~dlnorm(mm[i],taum)		# sampling to build model fit
```

```
		mm[i] <- m[1] + m[2]*temp[i] + m[3]*time1[i]+m[4]*time2[i]+m[5]*time3[i]+m[6]*time4[i]
+	m[7]*time1[i]*temp[i]+m[8]*time2[i]*temp[i]+m[9]*time3[i]*temp[i]+m[10]*time4[i]*temp[i]

		cpt1liver[i] ~dlnorm(mn[i],taun)  #log-normal distribution  (p=3)
		cpt1livermore[i] ~dlnorm(mn[i],taun)	# sampling to build model fit
		mn[i] <- n[1] + n[2]*temp[i] + n[3]*time1[i]+n[4]*time2[i]+n[5]*time1[i]*temp[i]
+n[6]*time2[i]*temp[i]

		random[i]~dlnorm(-0.08884447,3.886371)  # a random gene with average variance

	}
	for (i in 1:12) { # blocking information flow between weight and genes : we do not want weight
data to influence gene expression models.
		ac[i]<-cut(a[i]);bc[i]<-cut(b[i]);cc[i]<-cut(c[i]);dc[i]<-cut(d[i]);ec[i]<-cut(e[i]);fc[i]<-cut(f[i])
		gc[i]<-cut(g[i]);hc[i]<-cut(h[i]);mc[i]<-cut(m[i]);nc[i]<-cut(n[i])
	}
	# creating mean smooth gene expression dynamics at 9°C and 12°C
	for (i in 1:60) {
		ma9[i] <- ac[1]+ ac[3]*timep1[i]+ac[4]*timep2[i]
		ma12[i] <- ac[1]+ ac[2] + ac[3]*timep1[i]+ac[4]*timep2[i]+
ac[5]*timep1[i]+ac[6]*timep2[i]
		ma9p[i] ~dlnorm(ma9[i],taua)		# predictions at 9°C
		ma12p[i] ~dlnorm(ma12[i],taua)		# predictions at 12°C

		mb9[i] <- bc[1]+
bc[3]*timep1[i]+bc[4]*timep2[i]+bc[5]*timep3[i]+bc[6]*timep4[i]+bc[7]*timep5[i]
		mb12[i] <- bc[1]+bc[2]+
bc[3]*timep1[i]+bc[4]*timep2[i]+bc[5]*timep3[i]+bc[6]*timep4[i]+bc[7]*timep5[i]	+
bc[8]*timep1[i]+bc[9]*timep2[i]+bc[10]*timep3[i]+bc[11]*timep4[i]+bc[12]*timep5[i]
		mb9p[i]~dlnorm(mb9[i],taub)		# predictions at 9°C
		mb12p[i] ~dlnorm(mb12[i],taub)		# predictions at 12°C

		mc9[i] <- cc[1] + cc[2]*timep1[i] + cc[3]*timep2[i] + cc[4]*timep3[i]+
cc[5]*timep4[i]+cc[6]*timep5[i]+cc[7]*timep6[i]
		mc12[i]<-mc9[i]
		mc9p[i] ~dlnorm(mc9[i],tauc)		# predictions at 9°C
		mc12p[i] ~dlnorm(mc12[i],tauc)		# predictions at 12°C

		md9[i] <-dc[1] + dc[2]*timep1[i] + dc[3]*timep2[i] + dc[4]*timep3[i]+
dc[5]*timep4[i]+dc[6]*timep5[i]+dc[7]*timep6[i]
		md12[i]<-md9[i]
		md9p[i] ~dlnorm(md9[i],taud)		# predictions at 9°C
		md12p[i] ~dlnorm(md12[i],taud)		# predictions at 12°C

		me9[i] <- ec[1]+
ec[3]*timep1[i]+ec[4]*timep2[i]+ec[5]*timep3[i]+ec[6]*timep4[i]+ec[7]*timep5[i]
		me12[i] <-  ec[1]+ec[2]+
ec[3]*timep1[i]+ec[4]*timep2[i]+ec[5]*timep3[i]+ec[6]*timep4[i]+ec[7]*timep5[i]
	+ec[8]*timep1[i]+ec[9]*timep2[i]+ec[10]*timep3[i]+ec[11]*timep4[i]+ec[12]*timep5[i]
		me9p[i]~dlnorm(me9[i],taue)		# predictions at 9°C
		me12p[i] ~dlnorm(me12[i],taue)		# predictions at 12°C

		mf9[i] <- fc[1] + fc[3]*timep1[i] + fc[4]*timep2[i] + fc[5]*timep3[i]+
fc[6]*timep4[i]+fc[7]*timep5[i]+fc[8]*timep6[i]
		mf12[i] <- fc[1]+fc[2]+ fc[3]*timep1[i] + fc[4]*timep2[i] + fc[5]*timep3[i]+
fc[6]*timep4[i]+fc[7]*timep5[i]+fc[8]*timep6[i]
		mf9p[i] ~dlnorm(mf9[i],tauf)		# predictions at 9°C
		mf12p[i] ~dlnorm(mf12[i],tauf)		# predictions at 12°C
```

```
		mg9[i] <-gc[1] +  gc[2]*timep1[i] + gc[3]*timep2[i] + gc[4]*timep3[i]+ 
gc[5]*timep4[i]+gc[6]*timep5[i]+gc[7]*timep6[i]
		mg12[i]<-mg9[i]
		mg9p[i] ~dlnorm(mg9[i],taug)		# predictions at 9°C
		mg12p[i] ~dlnorm(mg12[i],taug)		# predictions at 12°C

		mh9[i] <-hc[1] + hc[2]*timep1[i] + hc[3]*timep2[i]
		mh12[i]<-mh9[i]
		mh9p[i] ~dlnorm(mh9[i],tauh)		# predictions at 9°C
		mh12p[i] ~dlnorm(mh12[i],tauh)		# predictions at 12°C

		mm9[i] <- mc[1]+ mc[3]*timep1[i]+mc[4]*timep2[i]+mc[5]*timep3[i]+mc[6]*timep4[i]
		mm12[i] <- mc[1]+mc[2]+ 
mc[3]*timep1[i]+mc[4]*timep2[i]+mc[5]*timep3[i]+mc[6]*timep4[i] + 
mc[7]*timep1[i]+mc[8]*timep2[i]+mc[9]*timep3[i]+mc[10]*timep4[i]
		mm9p[i] ~dlnorm(mm9[i],taum)		# predictions at 9°C
		mm12p[i] ~dlnorm(mm12[i],taum)		# predictions at 12°C

		mn9[i] <- nc[1]+ nc[3]*timep1[i]+nc[4]*timep2[i]
		mn12[i] <- nc[1]+ nc[2] + nc[3]*timep1[i]+nc[4]*timep2[i]+nc[5]*timep1[i]+nc[6]*timep2[i]
		mn9p[i] ~dlnorm(mn9[i],taun)		# predictions at 9°C
		mn12p[i] ~dlnorm(mn12[i],taun)		# predictions at 12°C

		# Creating cumulative for P(A), P(B), P(C) at 9°C
			#cumulative for autophagy
		h9gen_1234[i] <- sum(p9gen_1234[1:i]); p9gen_1234[i]<-
exp(ma9[i]+mb9[i]+mc9[i]+md9[i])
			#cumulative for gdh and cpt1
		h9gen_9[i] <- sum(p9gen_9[1:i]); p9gen_9[i]<-exp(mm9[i])
		h9gen_10[i] <- sum(p9gen_10[1:i]); p9gen_10[i]<-exp(mn9[i])

		# model for relative contributions of transcripts on weight loss at 9°C
		p9[i] <- p[1]*p9gen_1234[i]+ p[2]*p9gen_1234[i] *p9gen_9[i]+ p[3]*p9gen_1234[i] 
*p9gen_10[i]+ p[4]*p9gen_9[i] +p[5]*p9gen_10[i] +p[6]* cumulrandom [i]
		h9[i] <- sum(p9[1:i]);

		# Creating cumulative for P(A), P(B), P(C) at 12°C
			#cumulative for autophagy
		h12gen_1234[i] <- sum(p12gen_1234[1:i]); p12gen_1234[i]<-
exp(ma12[i]+mb12[i]+mc12[i]+md12[i])
			#cumulative for gdh and cpt1
		h12gen_9[i] <- sum(p12gen_9[1:i]); p12gen_9[i]<-exp(mm12[i])
		h12gen_10[i] <- sum(p12gen_10[1:i]); p12gen_10[i]<-exp(mn12[i])

		# model for relative contributions of transcripts on weight loss at 12°C
		p12[i] <- q[1]*p12gen_1234[i]+ q[2]*p12gen_1234[i] *p12gen_9[i]+ 
q[3]*p12gen_1234[i] *p12gen_10[i]+ q[4]*p12gen_9[i] +q[5]*p12gen_10[i] +q[6]* cumulrandom [i]
		h12[i] <- sum(p12[1:i]);

		# prediction for weight loss at 9°C
		weight9[i]<-alpha[1]*h9[i]
		tau1[i]<-1/exp(u1[1]+v1[1]*i)
		w9p[i]~dnorm(weight9[i],tau1[i])I(0,)

		# prediction for weight loss at 12°C
		weight12[i]<-alpha[2]*h12[i]
		tau2[i]<-1/exp(u2[1]+v2[1]*i)
		w12p[i]~dnorm(weight12[i],tau2[i])I(0,)

		# the cumulative of a random gene expression
```

```
		randomc[i]<-cut(random[i]); #random gene
		cumulrandom[i]<-sum(randomc[1:i])

	}
	# calculating differences of contributions between 9°C and 12°C
	for (i in 1:6) {     # with the random gene
		r[i]<-step(p[i]-q[i])
		}
	for (i in 1:5) {     # without the random gene
		s[i]<-step(pp[i]-qq[i])
	}
	# linking gene expression to individual weight loss
	for (i in 41:N) {
		timebis[i]<- timex[i]*10  # expanding time vector

		# Test model M1
		DPDS1[i]~dnorm(m1[i],taur1[i])
		# picking the right cumulative depending on temperature
		integ1[i]<- step(0-temp[i])* h9[timebis[i]] + step(temp[i]-1) * h12[timebis[i]]
		# actual inference of transcript cumulatives on weight loss
		m1[i]<- (step(0-temp[i])*alpha[1] + step(temp[i]-1) * alpha[2])*integ1[i]
		# variance is time and temperature dependent
		taur1[i]<-  step(0-temp[i])* (1/var11[i]) + step(temp[i]-1) * (1/var12[i])
		log(var11[i]) <- u1[1]+ v1[1]*timebis[i] ; log(var12[i]) <- u2[1]+ v2[1]*timebis[i]
		# sampling in distribution to estimate fit
		DPDS1more[i]~dnorm(m1[i],taur1[i])

		# Null Model M0
		DPDS2[i]~dnorm(m2[i],taur2[i])
		# actual inference of the random transcript cumulative on weight loss
		m2[i]<- (step(0-temp[i])*gamma[1] + step(temp[i]-1) * gamma[2])* cumulrandom
[timebis[i]]
		# variance is time and temperature dependent
		taur2[i]<-  step(0-temp[i])* (1/var21[i]) + step(temp[i]-1) * (1/var22[i])
		log(var21[i]) <- u1[2]+ v1[2]*timebis[i] ; log(var22[i]) <- u2[2]+ v2[2]*timebis[i]
		# sampling in distribution to estimate fit
		DPDS2more[i]~dnorm(m2[i],taur2[i])

	}




#priors
	# variance parameters priors
	taua<-1/vara; vara ~ dunif(0,100)
	taub<-1/varb; varb ~ dunif(0,100)
	tauc<-1/varc; varc ~ dunif(0,100)
	taud<-1/vard; vard ~ dunif(0,100)
	taue<-1/vare; vare ~ dunif(0,100)
	tauf<-1/varf; varf ~ dunif(0,100)
	taug<-1/varg; varg ~ dunif(0,100)
	tauh<-1/varh; varh ~ dunif(0,100)
	taum<-1/varm; varm ~ dunif(0,100)
	taun<-1/varn; varn ~ dunif(0,100)

	# weight loss models parameters
	for (i in 1:2) {
		alpha[i]~dunif(0,1000)
		gamma[i]~dunif(0,1000)
```

```
	}
	# parameters for time and temperature dependent variance.
	for (i in 1:24) {
		u1[i]~dnorm(0,0.001)
		u2[i]~dnorm(0,0.001)
		v1[i]~dnorm(0,0.001)
		v2[i]~dnorm(0,0.001)
	}

	# non informative priors for Dirichlet distribution.
	p[1:6] ~ ddirch(delta[])
	q[1:6] ~ ddirch(eta[])

	for(i in 1:6) {
		delta[i]<-1
		eta[i]<-1
	}

	# Scaling relative contributions to 1 without linear effect (random gene)
	for (i in 1:5) {
		pp[i]<- p[i]/(1-p[6])
		qq[i]<- q[i]/(1-q[6])
	}

	# gene expression models non informative priors.
	for (i in 1:14) {
		a[i]~dnorm(0, 0.001)
		b[i]~dnorm(0, 0.001)
		c[i]~dnorm(0, 0.001)
		d[i]~dnorm(0, 0.001)
		e[i]~dnorm(0, 0.001)
		f[i]~dnorm(0, 0.001)
		g[i]~dnorm(0, 0.001)
		h[i]~dnorm(0, 0.001)
		m[i]~dnorm(0, 0.001)
		n[i]~dnorm(0, 0.001)
	}
}
```

```
#################
### DATA ######
################


        #data
list(

ulk1=c(0.2694,1.1196,0.6634,0.4790,1.0021,0.6657,0.9031,0.9031,3.5504,0.7235,0.5775,0.8969,1.32
23,0.7594,2.4932,3.5876,0.5045,4.2960,NA,1.3500,0.2694,1.1196,0.6634,0.4790,1.0021,0.6657,0.90
31,0.9031,3.5504,0.7235,0.5775,0.8969,1.3223,0.7594,2.4932,3.5876,0.5045,4.2960,NA,1.3500,0.74
64,0.2990,0.5389,0.4890,0.3681,0.5559,0.3555,0.4070,0.2938,1.8830,0.2666,NA,0.7387,0.3850,0.40
56,0.3127,NA,0.7061,0.5045,0.2685,0.4840,NA,0.2021,0.1759,0.1821,NA,0.6276,0.4659,0.1495,0.27
22,0.4873,0.3904,0.2258,0.1815,0.2403,0.6750,0.2181,0.1591,0.1809,1.2509,0.4141,1.4825,0.3568,0
.6727,0.2838,1.1431,0.2959,0.4840,0.4907,0.1765,0.2918,0.9350,0.3837,0.1828,0.5370,0.4806,0.278
9,0.4084,0.2979,0.2354,0.3617,0.3306,0.9094,0.7464,0.1274,0.3375,0.3193,0.3824,0.2403,0.1419,0.
8604,0.5897,0.3084,0.2585,0.2722,0.3531,0.1986,0.5938,0.1688,0.3693,0.9917,0.3986,0.6342,0.166
4,0.1699,0.6169,0.2741,0.4773,0.3352,0.4000,0.3204,0.7387,2.3999,0.3052,0.4675,0.3732,0.5133,0.
9350,0.5559,0.8907,0.2657,0.3238,0.6126,0.4724,0.3681,0.9815,0.3758,0.7490,1.8764,0.5979,0.524
1,1.0267,1.1042),

cathf=c(0.4165,0.6461,1.2736,0.3409,1.7394,1.1153,0.5802,1.4966,13.1952,0.2421,1.4535,0.7440,1.
0958,0.5982,7.5994,2.8789,0.0855,2.9760,NA,1.2132,0.4165,0.6461,1.2736,0.3409,1.7394,1.1153,0.
5802,1.4966,13.1952,0.2421,1.4535,0.7440,1.0958,0.5982,7.5994,2.8789,0.0855,2.9760,NA,1.2132,3
.0150,0.3906,2.0084,0.4712,0.8767,1.1463,0.7731,1.7514,0.6912,3.9057,1.4666,NA,0.7299,NA,1.837
1,1.2724,0.9142,4.1435,1.4461,1.1659,0.6457,0.6693,0.9578,0.4954,0.4805,1.0057,2.2716,1.4240,0.
5788,0.7577,1.1628,2.0265,0.5541,0.4890,0.5329,1.5465,0.9647,0.5950,0.5685,6.7503,0.7867,2.774
4,1.2731,3.0560,1.5139,2.2582,0.9634,1.4156,2.4173,0.5114,0.8307,3.4041,1.0596,1.0366,2.2197,1.
3602,0.9359,1.7961,0.9268,0.7057,0.4594,0.7195,1.2683,2.0086,0.2819,0.8673,1.4049,1.2428,1.223
8,0.4323,2.1847,2.8176,0.3401,0.5463,0.7053,0.4625,0.6606,1.1498,0.2725,2.4340,3.0333,1.2820,1.
5328,0.8244,0.6727,3.2234,0.8318,2.0776,1.3727,0.9638,1.2936,1.8445,3.6837,0.5022,1.4979,1.071
4,2.8644,3.9938,1.9054,1.3256,0.9540,0.9690,1.0554,1.4110,1.2180,3.1968,1.5751,4.1164,2.6312,2.
2178,2.2293,3.9644,2.7055),

cathl=c(0.2573,1.4894,0.5858,0.9519,0.4535,0.5950,0.5228,NA,6.0392,0.7002,0.8261,1.1319,0.9257,
0.9427,3.9226,2.4895,0.4155,2.0169,0.3986,3.0733,0.2573,1.4894,0.5858,0.9519,0.4535,0.5950,0.52
28,NA,6.0392,0.7002,0.8261,1.1319,0.9257,0.9427,3.9226,2.4895,0.4155,2.0169,0.3986,3.0733,1.25
18,0.8497,0.3984,0.4904,0.6295,1.3936,0.6350,0.7178,0.5278,1.6494,0.5320,0.5493,0.4408,NA,0.82
72,0.6389,0.6430,0.7814,1.2690,0.5134,0.2475,NA,0.3797,0.2975,0.2666,0.9089,0.9335,0.5164,0.13
46,0.2406,0.2324,0.5620,NA,0.2044,0.2935,1.4240,0.3702,0.4197,0.3004,2.0707,0.4004,0.8469,0.30
28,0.6159,0.4828,1.2842,0.2334,0.5356,0.4245,0.3664,0.2756,1.3092,0.3498,0.1446,0.3990,0.2663,0
.1679,0.3292,0.2892,0.3082,0.1717,0.2348,0.2673,0.5249,0.2563,0.3068,0.5037,0.3972,0.2591,0.153
6,1.9146,0.7484,0.2506,0.1760,0.2359,0.2571,0.2249,0.2568,0.1256,0.3153,0.6551,0.3443,0.1780,0.
1224,0.2541,0.6634,0.1520,0.3201,0.1892,0.2347,0.4284,0.4565,0.4012,0.1458,0.3655,0.1915,0.496
8,0.4451,0.3617,0.4884,0.2899,0.5744,0.4009,0.3733,0.2450,1.0387,0.3563,0.8387,0.7365,0.3848,0.
3513,0.7447,0.5048),

lc3b=c(0.3859,1.1698,0.9010,0.7284,0.9322,0.7125,0.9577,1.0543,1.7546,0.9298,0.7705,1.2549,1.09
33,0.7982,2.2189,1.9024,0.4788,2.4467,NA,1.4115,0.3859,1.1698,0.9010,0.7284,0.9322,0.7125,0.95
77,1.0543,1.7546,0.9298,0.7705,1.2549,1.0933,0.7982,2.2189,1.9024,0.4788,2.4467,NA,1.4115,1.17
72,1.0032,1.6207,1.2421,0.8226,1.3262,0.8970,0.8457,0.7384,1.9227,0.8871,2.3573,0.8865,1.1857,0
.9418,0.8722,NA,1.6148,1.2356,0.8842,0.6514,0.7033,0.5101,0.4566,0.4125,0.7262,1.4350,0.6419,0.
3480,0.4981,0.4622,0.6482,0.4608,0.3815,0.5516,1.0205,0.4428,NA,0.4187,0.8578,0.4440,1.0427,0.
5122,1.0587,0.4521,1.0925,0.4264,1.0644,0.6359,0.5523,0.3756,1.0067,0.6016,0.3632,0.6420,0.584
3,0.4436,0.6451,0.4710,0.4499,0.4013,0.3433,0.6850,0.8391,0.3260,0.4152,0.4163,0.5390,0.3842,0.
2562,0.6280,0.6611,0.3473,0.2842,0.4646,0.5751,0.3200,0.5576,0.2988,0.5457,0.9726,0.4695,0.902
5,0.2759,0.2376,0.5565,0.3365,0.4320,0.3967,0.4810,0.5692,0.6179,1.5225,0.3185,0.5794,0.3279,0.
6993,0.8923,0.5400,0.7486,0.3637,0.4715,0.6180,0.4964,0.4357,1.0101,0.4780,0.6887,1.3900,0.648
8,0.6245,1.0368,0.9606),
```

```
cathd=c(0.3000,0.8973,1.1058,0.4633,1.0129,0.6965,0.6643,NA,18.9117,0.2190,NA,1.2735,1.1006,1.
8329,9.0648,5.4941,0.1934,4.3486,0.6271,NA,0.3000,0.8973,1.1058,0.4633,1.0129,0.6965,0.6643,N
A,18.9117,0.2190,NA,1.2735,1.1006,1.8329,9.0648,5.4941,0.1934,4.3486,0.6271,NA,4.0056,0.9654,
1.9390,1.2400,1.0344,1.8875,1.0325,1.4444,0.9138,3.5369,NA,1.8642,1.1176,1.2215,2.0157,2.8636,
NA,3.7095,2.7052,0.9956,0.6369,NA,0.9986,0.3562,NA,2.6825,3.0050,1.5503,0.6563,0.2063,0.3545,
2.2283,NA,0.7818,1.6493,2.8675,NA,1.3296,0.6152,12.9417,1.0094,4.4580,2.0801,2.7140,1.0070,3.0
782,1.1641,2.9567,2.3936,0.8115,2.2940,4.3684,1.5378,NA,3.3947,1.4310,1.2638,1.3887,2.0413,0.9
977,0.5889,NA,1.6097,2.4299,0.3851,1.5461,NA,1.4311,1.0280,0.4464,6.7794,4.1184,0.7351,0.5963,
0.8273,1.5012,0.4176,1.6287,0.8352,2.4268,4.6833,0.9071,2.7012,1.1370,0.3601,5.8751,1.3267,2.37
85,2.4160,1.7812,2.6449,3.9068,4.0008,1.2414,2.4727,1.2646,2.9421,NA,3.6462,2.3709,0.9770,3.15
78,3.0531,2.3109,2.3026,4.5264,1.9326,NA,6.6496,3.8865,4.1059,6.8016,4.4942),

atg7=c(0.8616,1.9465,0.6667,1.8487,0.7523,1.3426,2.1360,2.0174,1.0261,0.4575,1.3997,1.1450,1.03
02,0.2744,1.4886,1.1650,0.6723,0.6311,1.1158,0.6365,0.8616,1.9465,0.6667,1.8487,0.7523,1.3426,2
.1360,2.0174,1.0261,0.4575,1.3997,1.1450,1.0302,0.2744,1.4886,1.1650,0.6723,0.6311,1.1158,0.636
5,1.7997,0.7152,1.0303,1.0803,1.2992,1.7327,2.1527,1.4562,0.9239,1.2625,1.7488,1.5956,1.4899,0.
7678,1.7302,0.9097,0.7197,1.0264,1.8777,1.0820,0.6148,1.8349,0.8531,0.7806,0.3638,0.8588,0.957
1,1.0522,0.6631,0.4591,1.2418,1.0213,0.6817,0.6656,0.5795,1.4268,0.9391,1.7337,0.9827,0.2240,1.
1921,1.1966,1.4601,2.2320,0.8122,2.1933,0.8241,1.4398,1.3050,1.1131,1.1468,0.9034,1.6473,0.852
5,0.8670,1.7672,1.5357,1.2879,1.2517,1.1697,1.0166,0.5539,0.6614,1.5099,0.7600,0.9748,0.8357,1.
1369,0.1799,NA,1.3829,0.8770,0.7968,0.6932,0.4635,1.6069,1.6510,0.8045,0.3347,0.7402,1.2244,0.
9729,1.1335,0.7476,0.4817,0.7861,0.3776,0.4468,0.4390,1.8979,0.5216,0.4653,0.2336,0.5169,0.668
9,0.6370,0.8824,1.1728,0.4725,0.9890,1.0487,1.0833,0.4434,1.2101,0.8350,0.8660,0.6332,0.9942,1.
1153,0.6517,0.4991,0.6560,0.3205),

atg12=c(1.8914,1.7264,1.3661,0.6956,1.0740,1.6083,1.2721,0.6294,1.0709,0.6295,0.7148,0.9020,0.5
932,0.5814,0.8019,0.8589,2.0140,0.6024,2.0202,1.1865,1.8914,1.7264,1.3661,0.6956,1.0740,1.6083,
1.2721,0.6294,1.0709,0.6295,0.7148,0.9020,0.5932,0.5814,0.8019,0.8589,2.0140,0.6024,2.0202,1.18
65,0.5399,0.6120,0.7408,0.6345,0.5012,0.8056,0.6978,0.5251,0.7001,1.0102,0.6490,0.6522,0.7980,0
.3978,0.2877,0.4119,0.5112,0.7833,0.4174,0.4469,0.3928,0.6023,0.4187,0.5491,0.6359,0.3819,0.456
6,0.2481,0.3403,0.2439,1.7054,1.0615,0.3820,1.0022,0.8804,0.5754,1.1176,1.1191,0.8305,0.4142,0.
8468,1.4863,0.5628,0.9816,0.6172,1.1332,0.8627,1.1660,0.8212,1.0661,1.8412,1.1239,1.3640,0.798
3,0.9025,0.7631,0.7127,0.6937,1.3834,1.0511,1.0689,0.7381,2.0122,1.2073,1.0482,1.4233,1.2460,0.
8021,0.8189,NA,1.5387,1.1127,0.7106,0.6005,0.9803,1.9768,1.0769,0.8690,0.5732,1.3479,1.5653,1.
3483,2.2354,1.1616,0.9040,1.3404,1.0759,0.8403,1.0319,2.2814,1.1594,1.0113,2.0397,0.9087,0.715
4,1.3297,1.2137,1.7178,1.9310,1.7836,1.1026,1.5834,1.5871,1.7923,0.9694,2.7929,2.1437,1.7134,2.
3041,1.3860,1.7508,1.7185,1.7929),

catha=c(0.1436,2.3780,1.3372,1.4628,0.9987,0.8731,0.7753,0.9084,0.9090,0.4178,0.5299,0.6396,0.6
961,0.3304,0.5388,0.6429,0.2083,0.4023,0.2323,0.1864,0.1436,2.3780,1.3372,1.4628,0.9987,0.8731,
0.7753,0.9084,0.9090,0.4178,0.5299,0.6396,0.6961,0.3304,0.5388,0.6429,0.2083,0.4023,0.2323,0.18
64,1.2406,0.6219,1.2057,1.3562,0.7981,1.4642,1.0991,0.8069,0.8109,1.6244,1.0853,0.9003,1.2059,0
.5771,0.8237,0.6447,0.5820,0.6959,0.6506,0.8366,0.7672,1.1590,0.6304,0.9459,0.3098,0.5983,0.918
2,0.5641,0.3735,0.3972,1.6075,1.5290,0.7224,0.7184,1.4510,1.3321,1.5784,1.7343,0.7968,1.0874,1.
1624,1.5467,1.4824,2.2810,1.1461,2.2965,1.5237,1.3880,1.2641,1.5198,2.3592,2.5128,2.6692,1.029
8,2.1929,1.4138,1.5154,1.3465,1.3403,1.8566,1.0858,0.8872,0.6052,1.2897,1.1878,1.2843,0.9542,1.
0700,0.5998,NA,2.1471,0.9517,0.7334,0.7006,1.6787,2.5517,1.9113,1.0438,0.7245,1.1847,1.2137,1.
3919,1.5783,0.8127,0.6267,1.5484,1.1399,0.5139,0.6899,2.1750,1.3773,0.8063,0.8637,0.4692,0.882
3,0.7980,1.2047,1.6963,1.1917,1.0304,1.0765,1.7244,1.0205,1.0980,1.2199,2.3365,1.1252,1.6798,2.
1868,1.1571,1.1847,1.2776,1.5300),

cpt1liver=c(1.159238098,0.972078053,0.498755126,1.5363445,4.135353501,2.776859114,2.915833
089,1.73452993,5.378990217,3.085210186,2.888920074,3.252002176,2.573176662,2.96775141,4.2
46012182,4.740889755,2.009048603,3.058315265,2.507332361,1.473172732,1.159238098,0.97207
8053,0.498755126,1.5363445,4.135353501,2.776859114,2.915833089,1.73452993,5.378990217,3.0
85210186,2.888920074,3.252002176,2.573176662,2.96775141,4.246012182,4.740889755,2.009048
603,3.058315265,2.507332361,1.473172732,0.363153841,0.660190432,0.419721104,0.680751412,1
.611825618,1.244183818,0.816321232,0.429866616,0.783340531,0.735257838,2.011653829,0.9593
13575,0.81751134,2.219341835,0.523531331,1.234373108,0.896739607,1.810713755,0.780182113,
1.722906133,0.925805565,1.263433268,0.770163122,0.317261456,0.708773016,1.111396565,0.634
```

665563,0.337604222,NA,0.295543757,0.876509102,0.402138073,0.758156718,0.314961551,1.0817
97315,2.160104778,0.35910373,0.744801312,0.587143663,0.335019115,0.261157683,0.749582787,
1.06030362,0.627464786,0.630452275,0.348594014,0.524121407,0.241704343,0.552370585,0.6300
66505,0.186622712,0.686029873,0.259927631,0.176325499,0.418555264,0.832696941,0.24315400
3,0.484894811,0.402125359,0.264924754,1.388660009,0.42818755,0.697412893,0.34430536,0.535
282543,NA,0.681636106,0.430896776,0.361558711,0.550017009,0.461229538,1.117015465,0.4274
41345,0.490998126,0.370108939,0.609104529,0.637297139,0.241298382,0.116334268,0.32155587
2,0.17846486,0.188064348,0.46830757,0.53311046,0.137892615,0.261200454,0.384497504,0.4496
33897,0.226202221,0.112626891,0.237614489,0.231625386,0.426268747,0.667269985,0.56323920
2,0.419586039,0.447314268,0.130359812,0.329297294,0.341943967,0.439405514,0.537610113,0.3
28022355,0.297930396,0.322483032,0.409734981,0.381402096,0.217653509,0.290628052,0.37573
8979,0.176285978,0.328807517,0.167288175),

gdh=c(0.910266226,1.370029258,1.330072612,NA,0.610659854,0.783231521,1.543153871,0.71080
3567,1.483254478,1.618448122,0.855169161,0.545843539,0.78565705,0.399627827,1.212573651,1
.275459797,0.604611362,0.899757309,1.189680986,1.307326732,0.910266226,1.370029258,1.3300
72612,NA,0.610659854,0.783231521,1.543153871,0.710803567,1.483254478,1.618448122,0.85516
9161,0.545843539,0.78565705,0.399627827,1.212573651,1.275459797,0.604611362,0.899757309,1
.189680986,1.307326732,0.865174038,1.271091073,0.875181183,1.317495528,0.559839117,1.3000
2664,2.007770166,0.901556356,3.961755743,2.799254642,2.685681909,3.176908058,2.077071884,
2.752418426,3.217776394,1.782185791,2.274007729,1.302136431,2.536138319,3.055953879,1.082
540338,2.197680934,0.587044686,1.191004726,NA,1.442413652,1.006580951,4.572757293,1.4403
48155,0.791887781,0.778080241,1.949398904,2.215985579,1.054203807,1.510142453,2.25701007
1,1.565452922,1.117438322,2.497093381,0.981099832,0.57264786,1.057846911,0.917761926,1.00
0224853,2.283244389,1.348429254,1.809437611,1.041442558,0.691662312,0.487078993,1.045003
043,0.649980444,1.198980352,1.382535357,0.585034289,1.208534266,0.785537868,0.52608164,1.
8145969,0.682028798,0.366052124,1.163375098,0.740428876,3.407770272,NA,2.762048954,1.047
8826,1.05211858,2.626100069,0.765004325,0.748413472,0.763247079,1.386528515,0.510112941,0
.894260572,1.288364228,2.02216714,1.740088443,1.16055057,3.352206952,1.036741272,0.730625
543,0.649746738,0.836559675,0.678258479,0.347073529,0.752616069,1.318166161,0.497228834,0
.718563797,1.440837477,0.932481833,1.418922705,1.42149174,0.849591981,1.192105852,1.12398
7521,3.667901611,0.423199583,1.371709527,0.869671108,1.523783877,1.481911282,1.361506514,
1.426567074,1.829988415,0.624855632,0.915119359,0.926821601,0.666194618,2.226917116,1.698
313037,1.06980004),

time=c(0,0,0,0,0,0,0,0,0,0,0,0,0,0,0,0,0,0,0,0,0,0,0,0,0,0,0,0,0,0,0,0,0,0,0,0,0,0,0,0,10,10,10,10,10,10,
10,10,10,10,10,10,10,10,10,10,10,10,10,10,21,21,21,21,21,21,21,21,21,21,21,21,21,21,21,21,21,21,2
1,21,33,33,33,33,33,33,33,33,33,33,33,33,33,33,33,33,33,33,33,33,42,42,42,42,42,42,42,42,42,42,42,
42,42,42,42,42,42,42,42,53,53,53,53,53,53,53,53,53,53,53,53,53,53,53,53,53,53,53,60,60,60,60,60,6
0,60,60,60,60,60,60,60,60,60),

timex=c(0.0,0.0,0.0,0.0,0.0,0.0,0.0,0.0,0.0,0.0,0.0,0.0,0.0,0.0,0.0,0.0,0.0,0.0,0.0,0.0,0.0,0.0,0.0,0.0,0.0
,0.0,0.0,0.0,0.0,0.0,0.0,0.0,0.0,0.0,0.0,0.0,0.0,0.0,0.0,0.0,1.0,1.0,1.0,1.0,1.0,1.0,1.0,1.0,1.0,1.0,1.0,1.0,
1.0,1.0,1.0,1.0,1.0,1.0,1.0,1.0,2.1,2.1,2.1,2.1,2.1,2.1,2.1,2.1,2.1,2.1,2.1,2.1,2.1,2.1,2.1,2.1,2.1,2.1,2.1,
2.1,3.3,3.3,3.3,3.3,3.3,3.3,3.3,3.3,3.3,3.3,3.3,3.3,3.3,3.3,3.3,3.3,3.3,3.3,3.3,3.3,4.2,4.2,4.2,4.2,4.2,4.2,
4.2,4.2,4.2,4.2,4.2,4.2,4.2,4.2,4.2,4.2,4.2,4.2,4.2,5.3,5.3,5.3,5.3,5.3,5.3,5.3,5.3,5.3,5.3,5.3,5.3,5.3,5.3,
5.3,5.3,5.3,5.3,5.3,6.0,6.0,6.0,6.0,6.0,6.0,6.0,6.0,6.0,6.0,6.0,6.0,6.0,6.0,6.0
),

time1=c(-0.12883415, -0.12883415, -0.12883415, -0.12883415, -0.12883415, -0.12883415, -
0.12883415, -0.12883415, -0.12883415, -0.12883415, -0.12883415, -0.12883415, -0.12883415, -
0.12883415, -0.12883415, -0.12883415, -0.12883415, -0.12883415, -0.12883415, -0.12883415, -
0.12883415, -0.12883415, -0.12883415, -0.12883415, -0.12883415, -0.12883415, -0.12883415, -
0.12883415, -0.12883415, -0.12883415, -0.12883415, -0.12883415, -0.12883415, -0.12883415, -
0.12883415, -0.12883415, -0.12883415, -0.12883415, -0.12883415, -0.12883415, -0.08583555, -
0.08583555, -0.08583555, -0.08583555, -0.08583555, -0.08583555, -0.08583555, -0.08583555, -
0.08583555, -0.08583555, -0.08583555, -0.08583555, -0.08583555, -0.08583555, -0.08583555, -
0.08583555, -0.08583555, -0.08583555, -0.08583555, -0.08583555, -0.03853709, -0.03853709, -
0.03853709, -0.03853709, -0.03853709, -0.03853709, -0.03853709, -0.03853709, -0.03853709, -
0.03853709, -0.03853709, -0.03853709, -0.03853709, -0.03853709, -0.03853709, -0.03853709, -

0.03853709, -0.03853709, -0.03853709, -0.03853709, 0.01306123, 0.05175997, 0.05175997, 0.05175997, 0.05175997, 0.05175997, 0.05175997, 0.05175997, 0.05175997, 0.05175997, 0.05175997, 0.05175997, 0.05175997, 0.05175997, 0.05175997, 0.05175997, 0.05175997, 0.05175997, 0.05175997, 0.05175997, 0.09905843, 0.09905843, 0.09905843, 0.09905843, 0.09905843, 0.09905843, 0.09905843, 0.09905843, 0.09905843, 0.09905843, 0.09905843, 0.09905843, 0.09905843, 0.09905843, 0.09905843, 0.09905843, 0.09905843, 0.09905843, 0.09905843, 0.12915745, 0.12915745, 0.12915745, 0.12915745, 0.12915745, 0.12915745, 0.12915745, 0.12915745, 0.12915745, 0.12915745, 0.12915745, 0.12915745, 0.12915745, 0.12915745, 0.12915745),

time2=c(0.119781737,0.119781737, -0.005750497, -0.084902970, -0.100837687, -0.064570829, -0.064570829, -0.064570829, -0.064570829, -0.064570829, -0.064570829, -0.064570829, -0.064570829, -0.064570829, -0.064570829, -0.064570829, -0.064570829, -0.064570829, -0.064570829, -0.064570829, -0.064570829, -0.064570829, -0.064570829, -0.064570829, 0.035881982, 0.035881982, 0.035881982, 0.035881982, 0.035881982, 0.035881982, 0.035881982, 0.035881982, 0.035881982, 0.035881982, 0.035881982, 0.035881982, 0.035881982, 0.035881982, 0.035881982, 0.035881982, 0.035881982, 0.035881982, 0.035881982, 0.131951762, 0.131951762, 0.131951762, 0.131951762, 0.131951762, 0.131951762, 0.131951762, 0.131951762, 0.131951762, 0.131951762, 0.131951762, 0.131951762, 0.131951762, 0.131951762, 0.131951762),

time3=c(-0.09071397, -0.09071397, -0.09071397, -0.09071397, -0.09071397, -0.09071397, -0.09071397, -0.09071397, -0.09071397, -0.09071397, -0.09071397, -0.09071397, -0.09071397, -0.09071397, -0.09071397, -0.09071397, -0.09071397, -0.09071397, -0.09071397, -0.09071397,-0.09071397, 0.09930929, 0.09009204, -0.03298029, -0.10457231, -0.05376863, 0.11295577, 0.11295577, 0.11295577, 0.11295577, 0.11295577, 0.11295577, 0.11295577, 0.11295577, 0.11295577, 0.11295577, 0.11295577, 0.11295577, 0.11295577, 0.11295577, 0.11295577, 0.11295577),

time4=c(0.05127558, -0.12159435, 0.03977409, 0.09772114, -0.02145664, -0.12324355, -0.12324355, -0.12324355, -0.12324355, -0.12324355, -0.12324355, -0.12324355, -0.12324355, -0.12324355, -0.12324355, -0.12324355, -0.12324355, -0.12324355, -0.12324355, -0.12324355, -0.12324355, -0.12324355, -0.12324355, -0.12324355, 0.09371828),

time5=c(-0.02510176, 0.09541576, -0.12830179, 0.03097116, 0.09609736, -0.11133818, 0.05532721, 0.05532721, 0.05532721, 0.05532721, 0.05532721, 0.05532721, 0.05532721, 0.05532721, 0.05532721, 0.05532721, 0.05532721, 0.05532721, 0.05532721, 0.05532721, 0.05532721, 0.05532721),

time6=c(0.006855098,-0.036451410, 0.087336582, -0.143338880, -0.143338880, -0.143338880, -0.143338880, -0.143338880, -0.143338880, -0.143338880, -0.143338880, -0.143338880, -0.143338880, -0.143338880, -0.143338880, -

0.143338880, -0.143338880, -0.143338880, -0.143338880, -0.143338880, -0.143338880, -0.143338880, -0.143338880, 0.132792464, 0.132792464, 0.132792464, 0.132792464, 0.132792464, 0.132792464, 0.132792464, 0.132792464, 0.132792464, 0.132792464, 0.132792464, 0.132792464, 0.132792464, 0.132792464, 0.132792464, 0.132792464, 0.132792464, 0.132792464, 0.132792464, -0.059468183, -0.059468183, -0.059468183, -0.059468183, -0.059468183, -0.059468183, -0.059468183, -0.059468183, -0.059468183, -0.059468183, -0.059468183, -0.059468183, -0.059468183, -0.059468183, -0.059468183, -0.059468183, -0.059468183, -0.059468183, -0.059468183, 0.021254058, 0.021254058, 0.021254058, 0.021254058, 0.021254058, 0.021254058, 0.021254058, 0.021254058, 0.021254058, 0.021254058, 0.021254058, 0.021254058, 0.021254058, 0.021254058, 0.021254058),

timep1=c(-0.219910563, -0.212455968, -0.205001372, -0.197546777, -0.190092182, -0.182637586, -0.175182991, -0.167728396, -0.160273800, -0.152819205, -0.145364609, -0.137910014, -0.130455419, -0.123000823, -0.115546228, -0.108091633, -0.100637037, -0.093182442, -0.085727847, -0.078273251, -0.070818656, -0.063364061, -0.055909465, -0.048454870, -0.041000274, -0.033545679, -0.026091084, -0.018636488, -0.011181893, -0.003727298, 0.003727298, 0.011181893, 0.018636488, 0.026091084, 0.033545679, 0.041000274, 0.048454870, 0.055909465, 0.063364061, 0.070818656, 0.078273251, 0.085727847, 0.093182442, 0.100637037, 0.108091633, 0.115546228, 0.123000823, 0.130455419, 0.137910014, 0.145364609, 0.152819205, 0.160273800, 0.167728396, 0.175182991, 0.182637586, 0.190092182, 0.197546777, 0.205001372, 0.212455968, 0.219910563),

timep2=c(0.274592466, 0.246667808, 0.219706070, 0.193707251, 0.168671351, 0.144598370, 0.121488309, 0.099341167, 0.078156944, 0.057935640, 0.038677256, 0.020381790, 0.003049244, -0.013320383, -0.028727090, -0.043170879, -0.056651748, -0.069169698, -0.080724728, -0.091316840, -0.100946032, -0.109612305, -0.117315659, -0.124056094, -0.129833609, -0.134648205, -0.138499882, -0.141388640, -0.143314478, -0.144277397, -0.144277397, -0.143314478, -0.141388640, -0.138499882, -0.134648205, -0.129833609, -0.124056094, -0.117315659, -0.109612305, -0.100946032, -0.091316840, -0.080724728, -0.069169698, -0.056651748, -0.043170879, -0.028727090, -0.013320383, 0.003049244, 0.020381790, 0.038677256, 0.057935640, 0.078156944, 0.099341167, 0.121488309, 0.144598370, 0.168671351, 0.193707251, 0.219706070, 0.246667808, 0.274592466),

timep3=c(-0.309043624, -0.246187294, -0.188749613, -0.136540453, -0.089369686, -0.047047184, -0.009382819, 0.023813537, 0.052732012, 0.077562734, 0.098495832, 0.115721432, 0.129429664, 0.139810655, 0.147054533, 0.151351427, 0.152891464, 0.151864773, 0.148461481, 0.142871716, 0.135285608, 0.125893282, 0.114884869, 0.102450495, 0.088780289, 0.074064378, 0.058492892, 0.042255957, 0.025543702, 0.008546255, -0.008546255, -0.025543702, -0.042255957, -0.058492892, -0.074064378, -0.088780289, -0.102450495, -0.114884869, -0.125893282, -0.135285608, -0.142871716, -0.148461481, -0.151864773, -0.152891464, -0.151351427, -0.147054533, -0.139810655, -0.129429664, -0.115721432, -0.098495832, -0.077562734, -0.052732012, -0.023813537, 0.009382819, 0.047047184, 0.089369686, 0.136540453, 0.188749613, 0.246187294, 0.309043624),

timep4=c(0.327790264, 0.216674920, 0.122801612, 0.044758712, -0.018814994, -0.069230304, -0.107747601, -0.135576852, -0.153877610, -0.163759010, -0.166279776, -0.162448212, -0.153222211, -0.139509246, -0.122166380, -0.102000256, -0.079767104, -0.056172739, -0.031872559, -0.007471549, 0.016475723, 0.039465105, 0.061042858, 0.080805659, 0.098400602, 0.113525195, 0.125927362, 0.135405440, 0.141808184, 0.145034764, 0.145034764, 0.141808184, 0.135405440, 0.125927362, 0.113525195, 0.098400602, 0.080805659, 0.061042858, 0.039465105, 0.016475723, -0.007471549, -0.031872559, -0.056172739, -0.079767104, -0.102000256, -0.122166380, -0.139509246, -0.153222211, -0.162448212, -0.166279776, -0.163759010, -0.153877610, -0.135576852, -0.107747601, -0.069230304, -0.018814994, 0.044758712, 0.122801612, 0.216674920, 0.327790264),

timep5=c(-0.33334643, -0.16384824, -0.03526342, 0.05815028, 0.12167365, 0.16014283, 0.17796610, 0.17914065, 0.16726934, 0.14557753, 0.11692979, 0.08384674, 0.04852180, 0.01283797, -0.02161538, -0.05352574, -0.08184069, -0.10575109, -0.12467434, -0.13823755, -0.14626081, -0.14874041, -0.14583201, -0.13783391, -0.12517026, -0.10837425, -0.08807138, -0.06496265, -0.03980779, -0.01340847, 0.01340847, 0.03980779, 0.06496265, 0.08807138,

0.10837425, 0.12517026, 0.13783391, 0.14583201, 0.14874041, 0.14626081, 0.13823755, 0.12467434, 0.10575109, 0.08184069, 0.05352574, 0.02161538, -0.01283797, -0.04852180, -0.08384674, -0.11692979, -0.14557753, -0.16726934, -0.17914065, -0.17796610, -0.16014283, -0.12167365, -0.05815028, 0.03526342, 0.16384824, 0.33334643),

timep6=c(0.32779026, 0.09444804, -0.05843134, -0.14778744, -0.18829110, -0.19252599, -0.17116334, -0.13313003, -0.08576989, -0.03499831, 0.01454986, 0.05937915, 0.09699906, 0.12579628, 0.14491377, 0.15413641, 0.15378350, 0.14460791, 0.12770198, 0.10441011, 0.07624812, 0.04482929, 0.01179718, -0.02123493, -0.05273778, -0.08131317, -0.10573435, -0.12497955, -0.13825895, -0.14503476, -0.14503476, -0.13825895, -0.12497955, -0.10573435, -0.08131317, -0.05273778, -0.02123493, 0.01179718, 0.04482929, 0.07624812, 0.10441011, 0.12770198, 0.14460791, 0.15378350, 0.15413641, 0.14491377, 0.12579628, 0.09699906, 0.05937915, 0.01454986, -0.03499831, -0.08576989, -0.13313003, -0.17116334, -0.19252599, -0.18829110, -0.14778744, -0.05843134, 0.09444804, 0.32779026),

DPDS1=c(0,0,0,0,0,0,0,0,0,0,0,0,0,0,0,0,0,0,0,0,0,0,0,0,0,0,0,0,0,0,0,0,0,0,0,0,0,0,0,0,11,17,18,16,21,25,24,26,15,18,20,14,29,17,9,18,23,20,14,20,31,31,23,24,28,25,48,34,36,22,24,31,40,36,29,30,27,29,25,29,30,48,24,53,22,42,29,34,29,35,42,44,57,33,38,48,55,38,35,51,37,60,35,42,40,59,37,41,53,43,74,74,74,73,91,70,53,47,54,48,47,61,98,55,53,47,55,42,62,60,74,119,103,51,70,79,98,69,59,88,64,67,58,62,61,90,53,70,104,73,62,82,153),

DPDS2=c(0,0,0,0,0,0,0,0,0,0,0,0,0,0,0,0,0,0,0,0,0,0,0,0,0,0,0,0,0,0,0,0,0,0,0,0,0,0,0,0,11,17,18,16,21,25,24,26,15,18,20,14,29,17,9,18,23,20,14,20,31,31,23,24,28,25,48,34,36,22,24,31,40,36,29,30,27,29,25,29,30,48,24,53,22,42,29,34,29,35,42,44,57,33,38,48,55,38,35,51,37,60,35,42,40,59,37,41,53,43,74,74,74,73,91,70,53,47,54,48,47,61,98,55,53,47,55,42,62,60,74,119,103,51,70,79,98,69,59,88,64,67,58,62,61,90,53,70,104,73,62,82,153),

temp=c(0,0,0,0,0,0,0,0,0,0,0,0,0,0,0,0,0,0,0,0,1,1,1,1,1,1,1,1,1,1,1,1,1,1,1,1,1,1,1,1,0,0,0,0,0,0,0,0,0,0,1,1,1,1,1,1,1,1,1,1,0,0,0,0,0,0,0,0,0,0,1,1,1,1,1,1,1,1,1,1,0,0,0,0,0,0,0,0,0,0,1,1,1,1,1,1,1,1,1,1,0,0,0,0,0,0,0,0,0,0,1,1,1,1,1,1,1,1,1,0,0,0,0,0,0,0,0,0,0,1,1,1,1,1,1,1,1,1,0,0,0,0,0,0,0,0,0,0,1,1,1,1,1),

N=153)

```
####################

####################
# INITS EXAMPLES  ##
###################
list(
DPDS1more = c(
	NA,	NA,	NA,	NA,	NA,
	NA,	NA,	NA,	NA,	NA,
	NA,	NA,	NA,	NA,	NA,
	NA,	NA,	NA,	NA,	NA,
	NA,	NA,	NA,	NA,	NA,
	NA,	NA,	NA,	NA,	NA,
	NA,	NA,	NA,	NA,	NA,
	NA,	NA,	NA,	NA,	NA,
-1.85,7.28,0.05737,3.375,2.207,
2.02,15.58,0.5206,-1.733,0.4019,
24.45,16.9,15.86,24.96,24.1,
20.13,19.65,24.28,21.5,17.97,
26.42,20.62,17.61,16.94,34.72,
0.9315,21.19,14.46,31.47,31.39,
33.41,26.75,24.46,31.71,31.5,
31.22,32.84,33.59,36.42,30.14,
16.36,43.29,42.7,46.9,27.29,
16.68,26.56,47.1,40.64,45.19,
26.02,63.98,54.1,43.85,43.42,
42.29,59.61,55.97,44.31,32.73,
64.15,40.5,67.21,32.14,78.73,
60.36,-0.4184,49.08,53.36,32.22,
75.72,65.6,72.28,73.06,48.01,
64.16,65.18,52.24,46.41,91.96,
78.23,73.12,43.54,77.34,120.0,
23.45,19.4,107.1,47.15,116.4,
101.9,115.2,76.21,120.8,57.1,
85.66,98.41,50.05,26.57,17.13,
97.17,128.1,67.53,92.18,88.36,
79.88,142.2,96.96,111.4,117.4,
59.81,72.54,86.75),
DPDS2more = c(
	NA,	NA,	NA,	NA,	NA,
	NA,	NA,	NA,	NA,	NA,
	NA,	NA,	NA,	NA,	NA,
	NA,	NA,	NA,	NA,	NA,
	NA,	NA,	NA,	NA,	NA,
	NA,	NA,	NA,	NA,	NA,
	NA,	NA,	NA,	NA,	NA,
	NA,	NA,	NA,	NA,	NA,
20.42,6.226,6.832,8.861,13.03,
12.85,16.59,11.44,8.902,14.54,
20.68,11.25,12.22,11.57,6.45,
3.587,7.859,21.85,14.41,13.71,
23.11,16.26,9.082,25.79,17.7,
21.96,10.98,13.0,4.174,13.0,
25.2,20.44,39.82,16.9,37.5,
36.01,27.43,26.54,16.99,35.57,
36.57,33.55,48.05,59.33,46.02,
31.13,57.9,21.78,31.16,34.1,
24.69,20.2,26.79,33.02,43.44,
36.25,38.38,58.05,33.9,45.95,
38.49,45.68,42.35,40.19,58.07,
48.68,65.98,41.12,61.01,53.66,
33.18,34.22,29.24,44.03,56.88,
53.35,86.09,44.85,63.01,68.58,
41.44,37.79,64.41,47.84,50.69,
53.32,42.98,36.5,61.0,87.48,
45.48,109.7,53.19,80.62,100.8,
```

```
58.24,18.63,56.56,73.3,43.64,
41.23,29.91,52.79,69.93,78.77,
70.81,81.99,51.45,69.21,57.95,
77.56,70.56,104.6),
DPDSmorebis = c(
        NA,     NA,     NA,     NA,     NA,
        NA,     NA,     NA,     NA,     NA,
        NA,     NA,     NA,     NA,     NA,
        NA,     NA,     NA,     NA,     NA,
        NA,     NA,     NA,     NA,     NA,
        NA,     NA,     NA,     NA,     NA,
        NA,     NA,     NA,     NA,     NA,
        NA,     NA,     NA,     NA,     NA,
3.771,4.508,3.974,3.031,3.655,
3.866,4.379,4.207,3.704,3.803,
3.36,3.488,4.282,3.437,3.354,
3.926,3.982,4.18,4.079,4.501,
4.152,3.674,4.099,3.411,3.561,
3.009,4.125,3.636,3.904,4.492,
4.585,4.023,3.12,3.719,3.66,
3.143,3.112,4.791,3.437,3.886,
4.792,3.523,2.924,3.579,3.02,
3.703,4.252,4.351,4.964,3.68,
3.45,3.147,4.847,4.426,2.521,
4.0,3.544,3.276,3.472,3.354,
4.569,3.255,3.591,3.854,4.063,
3.452,4.571,3.491,3.82,3.581,
2.967,3.016,3.547,3.202,3.642,
3.159,3.607,3.701,3.994,3.288,
3.509,5.36,2.961,3.969,3.795,
3.954,3.903,4.073,3.747,3.965,
3.693,2.39,4.128,3.724,4.047,
3.487,3.409,3.768,3.056,3.334,
2.763,3.482,3.518,3.886,3.027,
3.33,3.451,3.271,4.294,4.726,
3.651,4.95,3.631),
a = c(
-0.7963,0.05529,-1.101,3.269,-0.8505,
1.231,-40.3,-10.39,-46.16,-35.31,
-77.54,-40.67,5.728,5.315),
alpha = c(
0.1708,0.4825),
atg12 = c(
        NA,     NA,     NA,     NA,     NA,
        NA,     NA,     NA,     NA,     NA,
        NA,     NA,     NA,     NA,     NA,
        NA,     NA,     NA,     NA,     NA,
        NA,     NA,     NA,     NA,     NA,
        NA,     NA,     NA,     NA,     NA,
        NA,     NA,     NA,     NA,     NA,
        NA,     NA,     NA,     NA,     NA,
        NA,     NA,     NA,     NA,     NA,
        NA,     NA,     NA,     NA,     NA,
        NA,     NA,     NA,     NA,     NA,
        NA,     NA,     NA,     NA,     NA,
        NA,     NA,     NA,     NA,     NA,
        NA,     NA,     NA,     NA,     NA,
        NA,     NA,     NA,     NA,     NA,
        NA,     NA,     NA,     NA,     NA,
        NA,     NA,     NA,     NA,     NA,
        NA,     NA,     NA,     NA,     NA,
        NA,     NA,     NA,     NA,     NA,
        NA,     NA,     NA,     NA,     NA,
        NA,     NA,     NA,     NA,     NA,
        NA,     NA,     NA,     NA,1.273,
        NA,     NA,     NA,     NA,     NA,
        NA,     NA,     NA,     NA,     NA,
```

```
        NA,     NA,     NA,     NA,     NA,
        NA,     NA,     NA,     NA,     NA,
        NA,     NA,     NA,     NA,     NA,
        NA,     NA,     NA,     NA,     NA,
        NA,     NA,     NA,     NA,     NA,
        NA,     NA,     NA,     NA,     NA,
        NA,     NA,     NA),
atg12more = c(
0.8247,1.531,0.7302,0.8428,0.5108,
0.8189,0.9319,1.102,0.5532,1.17,
0.7458,0.5446,0.4527,0.6012,0.674,
0.8821,1.44,0.8056,1.313,0.582,
0.6205,0.9331,1.301,1.094,1.105,
1.148,0.9749,0.9233,1.259,1.256,
1.047,0.7825,2.223,1.113,0.9872,
1.169,1.233,0.8217,0.9433,1.022,
0.7521,0.5185,0.648,0.5646,0.8381,
0.7201,0.7329,0.5849,0.7852,0.4538,
0.6237,0.4822,0.8614,0.3417,0.5901,
1.097,0.5661,0.5994,0.6571,0.6128,
0.4158,0.5844,0.2013,0.5533,0.6626,
0.7791,0.4895,0.331,0.3565,0.3067,
1.272,0.7804,0.9144,1.001,0.9866,
0.8782,1.644,0.8763,0.8279,0.9088,
1.268,1.715,1.119,0.8427,1.19,
1.356,0.6683,1.096,1.061,0.7537,
1.197,0.8766,0.9798,1.495,0.8744,
1.313,0.5403,0.8543,0.552,0.6557,
1.151,0.8464,1.738,0.8548,3.012,
0.7768,0.9803,1.992,0.8441,1.388,
1.643,1.339,0.7589,0.5719,1.089,
1.073,0.692,0.544,0.856,1.284,
0.5444,1.035,0.6351,1.039,0.5906,
1.1,1.619,0.9952,1.839,2.684,
1.445,1.683,1.366,1.689,1.505,
1.952,1.513,1.225,1.465,2.008,
1.43,1.311,1.927,1.573,2.09,
1.856,1.241,1.215,1.557,2.545,
1.146,1.875,1.759),
atg7 = c(
        NA,     NA,     NA,     NA,     NA,
        NA,     NA,     NA,     NA,     NA,
        NA,     NA,     NA,     NA,     NA,
        NA,     NA,     NA,     NA,     NA,
        NA,     NA,     NA,     NA,     NA,
        NA,     NA,     NA,     NA,     NA,
        NA,     NA,     NA,     NA,     NA,
        NA,     NA,     NA,     NA,     NA,
        NA,     NA,     NA,     NA,     NA,
        NA,     NA,     NA,     NA,     NA,
        NA,     NA,     NA,     NA,     NA,
        NA,     NA,     NA,     NA,     NA,
        NA,     NA,     NA,     NA,     NA,
        NA,     NA,     NA,     NA,     NA,
        NA,     NA,     NA,     NA,     NA,
        NA,     NA,     NA,     NA,     NA,
        NA,     NA,     NA,     NA,     NA,
        NA,     NA,     NA,     NA,     NA,
        NA,     NA,     NA,     NA,     NA,
        NA,     NA,     NA,     NA,     NA,
        NA,     NA,     NA,     NA,     NA,
        NA,     NA,     NA,     NA,0.7962,
        NA,     NA,     NA,     NA,     NA,
        NA,     NA,     NA,     NA,     NA,
        NA,     NA,     NA,     NA,     NA,
        NA,     NA,     NA,     NA,     NA,
        NA,     NA,     NA,     NA,     NA,
```

```
	NA,	NA,	NA,	NA,	NA,
	NA,	NA,	NA,	NA,	NA,
	NA,	NA,	NA,	NA,	NA,
	NA,	NA,	NA),
atg7more = c(
1.28,1.659,0.9564,0.5031,1.577,
2.395,1.025,0.9149,0.9367,0.5339,
2.876,1.024,0.8352,0.9397,0.5959,
0.7773,0.8267,3.894,0.9556,1.594,
1.053,1.106,1.659,1.156,1.122,
1.229,1.04,1.192,0.6783,0.82,
0.4794,0.7101,2.083,1.799,0.6833,
1.225,0.7517,1.626,1.039,1.099,
0.6349,0.7709,1.143,0.5876,0.8841,
1.194,1.745,2.016,0.7703,0.4315,
1.569,0.9781,2.821,1.672,1.397,
1.93,0.8748,2.182,2.166,4.764,
0.7389,1.37,0.221,0.6085,0.4568,
0.4899,0.7131,0.5708,0.3689,1.72,
1.117,0.3608,0.9384,1.154,0.3563,
0.3621,0.8771,0.4437,0.3932,0.478,
0.9071,3.364,0.7495,0.4034,2.373,
0.612,1.661,0.8408,1.764,0.5208,
0.5809,1.528,0.9765,2.685,1.403,
1.066,1.874,1.954,5.615,0.7468,
0.4571,0.4123,1.377,0.6793,1.105,
0.6689,1.262,0.3098,0.7352,0.6099,
1.516,0.861,0.3779,0.8022,2.588,
1.264,0.7223,0.6913,0.905,0.7302,
0.3256,0.3577,0.2958,0.5461,0.5771,
0.5225,0.6063,2.171,0.5636,1.776,
1.158,0.4057,0.297,0.8761,0.4401,
0.4045,1.986,0.8508,1.628,0.6756,
0.5453,2.799,1.157,0.87,1.005,
0.4607,0.8026,0.5979,0.394,1.073,
0.6536,0.3548,0.698),
b = c(
-0.162,0.1638,3.206,1.799,-1.821,
0.3664,1.652,-0.1348,0.2945,0.8034,
0.8768,-3.708,2.379,-26.76),
c = c(
-0.3731,-2.758,1.661,0.7354,-0.8033,
0.5875,-0.7488,-41.69,5.948,14.6,
7.53,61.65,-5.264,0.3017),
catha = c(
	NA,	NA,	NA,	NA,	NA,
	NA,	NA,	NA,	NA,	NA,
	NA,	NA,	NA,	NA,	NA,
	NA,	NA,	NA,	NA,	NA,
	NA,	NA,	NA,	NA,	NA,
	NA,	NA,	NA,	NA,	NA,
	NA,	NA,	NA,	NA,	NA,
	NA,	NA,	NA,	NA,	NA,
	NA,	NA,	NA,	NA,	NA,
	NA,	NA,	NA,	NA,	NA,
	NA,	NA,	NA,	NA,	NA,
	NA,	NA,	NA,	NA,	NA,
	NA,	NA,	NA,	NA,	NA,
	NA,	NA,	NA,	NA,	NA,
	NA,	NA,	NA,	NA,	NA,
	NA,	NA,	NA,	NA,	NA,
	NA,	NA,	NA,	NA,	NA,
	NA,	NA,	NA,	NA,	NA,
	NA,	NA,	NA,	NA,	NA,
	NA,	NA,	NA,	NA,	NA,
	NA,	NA,	NA,	NA,	NA,
	NA,	NA,	NA,	NA,1.4,
```

```
            NA,           NA,           NA,           NA,           NA,
            NA,           NA,           NA,           NA,           NA,
            NA,           NA,           NA,           NA,           NA,
            NA,           NA,           NA,           NA,           NA,
            NA,           NA,           NA,           NA,           NA,
            NA,           NA,           NA,           NA,           NA,
            NA,           NA,           NA,           NA,           NA,
            NA,           NA,           NA,           NA,           NA,
            NA,           NA,           NA),
cathamore = c(
0.9626,1.072,0.19,0.6272,0.318,
0.6754,0.7044,0.5241,0.3983,0.5135,
0.2985,0.9622,0.4524,0.3346,0.6839,
0.5265,0.8257,0.6617,0.3896,0.6542,
0.2826,1.204,0.4649,0.5213,0.8513,
0.5153,0.3085,0.5161,0.5979,0.2909,
0.2889,0.3861,0.4474,0.7266,1.236,
0.7544,0.2434,0.2785,0.6343,0.7513,
1.306,1.198,1.59,1.429,1.47,
1.094,2.276,1.054,0.5141,1.025,
1.112,1.458,1.895,0.3516,1.588,
0.9343,0.5296,0.7286,0.4535,0.8154,
0.7963,1.492,0.4776,0.4559,0.9313,
1.436,0.6411,1.082,1.424,0.4052,
3.336,2.124,1.101,2.255,1.533,
3.866,1.393,1.989,1.365,2.391,
1.116,1.151,1.898,1.071,0.831,
1.509,1.247,1.841,3.855,1.117,
1.028,0.8896,0.7277,2.366,1.085,
0.8651,1.664,1.081,1.508,1.649,
1.244,0.4716,2.042,1.073,1.112,
0.8979,1.442,1.944,0.9448,1.084,
2.831,0.8612,0.7115,2.226,1.456,
0.8659,1.145,0.9945,1.544,0.399,
0.5961,2.365,1.281,0.4382,0.7262,
0.9379,0.8695,0.4981,0.5086,1.946,
0.9834,1.442,1.436,0.9987,1.061,
1.326,0.3997,1.358,0.6174,1.863,
1.129,0.8081,1.126,2.198,0.8258,
1.374,2.829,1.211,0.6673,0.7795,
1.093,1.743,1.36),
cathd = c(
            NA,           NA,           NA,           NA,           NA,
            NA,           NA,1.442,           NA,           NA,
0.4241,           NA,           NA,           NA,           NA,
            NA,           NA,           NA,           NA,2.142,
            NA,           NA,           NA,           NA,           NA,
            NA,           NA,0.5091,           NA,           NA,
1.321,           NA,           NA,           NA,           NA,
            NA,           NA,           NA,           NA,0.8135,
            NA,           NA,           NA,           NA,           NA,
            NA,           NA,           NA,           NA,           NA,
8.792,           NA,           NA,           NA,           NA,
            NA,1.593,           NA,           NA,           NA,
            NA,0.08459,           NA,           NA,1.242,
            NA,           NA,           NA,           NA,           NA,
            NA,           NA,0.9741,           NA,           NA,
            NA,1.055,           NA,           NA,           NA,
            NA,           NA,           NA,           NA,           NA,
            NA,           NA,           NA,           NA,           NA,
            NA,           NA,           NA,1.063,           NA,
            NA,           NA,           NA,           NA,           NA,
            NA,0.3811,           NA,           NA,           NA,
            NA,0.5364,           NA,           NA,           NA,
            NA,           NA,           NA,           NA,           NA,
            NA,           NA,           NA,           NA,           NA,
            NA,           NA,           NA,           NA,           NA,
```

```
        NA,         NA,         NA,         NA,         NA,
        NA,         NA,         NA,         NA,         NA,
        NA,         NA,2.837,         NA,         NA,
        NA,         NA,         NA,         NA,         NA,
        NA,         NA,1.492,         NA,         NA,
        NA,         NA,         NA),
cathdmore = c(
0.9926,1.639,3.52,1.033,0.579,
0.9877,0.4746,0.5779,0.5639,2.062,
2.769,2.681,0.7392,2.608,3.741,
1.736,0.6931,1.303,0.8631,1.346,
0.68,1.368,1.272,0.3885,1.567,
0.3933,1.531,1.701,1.978,0.5929,
1.707,0.6308,4.613,1.064,0.7241,
0.4853,0.8765,2.812,0.5405,0.5792,
0.1223,5.314,1.671,3.112,3.252,
2.831,0.8063,3.873,4.487,0.3946,
2.877,1.615,12.45,1.428,3.717,
1.744,4.484,0.56,0.7805,3.733,
0.7495,3.076,0.4931,1.73,0.736,
3.443,1.833,0.675,3.061,0.3371,
1.432,0.4274,1.335,0.4089,0.4309,
0.2438,5.647,0.6596,0.9374,1.245,
1.389,2.694,2.982,0.9884,1.126,
1.786,0.6092,1.475,0.2621,1.955,
1.907,2.041,3.905,0.926,1.452,
0.549,9.179,0.4316,11.19,0.4218,
1.347,3.165,0.5009,2.04,5.971,
1.587,2.055,14.0,1.174,2.679,
2.954,1.122,2.105,1.602,0.7722,
0.8016,2.622,0.9203,1.447,3.46,
2.715,1.404,1.397,2.122,7.608,
0.9777,1.459,2.815,5.665,0.65,
1.167,3.562,4.951,1.271,2.363,
0.2679,2.743,0.8712,0.4509,3.704,
2.658,1.658,17.94,6.202,5.686,
1.358,1.738,6.039,1.382,3.38,
2.21,1.489,8.234),
cathf = c(
        NA,         NA,         NA,         NA,         NA,
        NA,         NA,         NA,         NA,         NA,
        NA,         NA,         NA,         NA,         NA,
        NA,         NA,         NA,1.036,         NA,
        NA,         NA,         NA,         NA,         NA,
        NA,         NA,         NA,         NA,         NA,
        NA,         NA,         NA,         NA,         NA,
        NA,         NA,         NA,0.8819,         NA,
        NA,         NA,         NA,         NA,         NA,
        NA,         NA,         NA,         NA,         NA,
        NA,0.7692,         NA,1.822,         NA,
        NA,         NA,         NA,         NA,         NA,
        NA,         NA,         NA,         NA,         NA,
        NA,         NA,         NA,         NA,         NA,
        NA,         NA,         NA,         NA,         NA,
        NA,         NA,         NA,         NA,         NA,
        NA,         NA,         NA,         NA,         NA,
        NA,         NA,         NA,         NA,         NA,
        NA,         NA,         NA,         NA,         NA,
        NA,         NA,         NA,         NA,         NA,
        NA,         NA,         NA,         NA,         NA,
        NA,         NA,         NA,         NA,         NA,
        NA,         NA,         NA,         NA,         NA,
        NA,         NA,         NA,         NA,         NA,
        NA,         NA,         NA,         NA,         NA,
        NA,         NA,         NA,         NA,         NA,
        NA,         NA,         NA,         NA,         NA,
```

```
          NA,          NA,          NA,          NA,          NA,
          NA,          NA,          NA,          NA,          NA,
          NA,          NA,          NA),
cathfmore = c(
0.6755,0.7191,0.6148,0.5831,0.4895,
1.744,0.7654,2.641,0.5963,0.5833,
1.967,3.244,2.135,3.388,0.5808,
0.6517,1.163,2.811,1.786,2.523,
0.9216,0.6493,0.4292,1.07,1.862,
0.4011,1.566,2.027,1.384,0.6734,
0.2056,0.6234,1.447,0.2844,1.113,
4.324,0.9133,0.3416,2.589,1.842,
0.8855,0.303,11.45,0.5422,0.3811,
1.213,0.8924,0.5348,1.104,0.9718,
1.284,0.9729,1.241,0.7998,2.068,
2.294,0.6131,1.849,1.892,0.8582,
3.255,0.9558,1.031,1.093,0.4026,
0.7271,1.124,0.2924,1.206,1.374,
0.4186,0.9997,0.5397,0.2593,1.479,
2.567,0.2184,1.156,0.3815,3.006,
0.8183,2.786,2.81,1.368,1.306,
1.287,1.836,1.064,3.562,2.1,
1.645,0.8144,0.6341,1.344,1.085,
0.8703,3.507,1.366,0.9118,2.737,
0.2751,6.624,2.483,1.163,2.924,
1.298,0.7287,0.8133,0.9381,0.5161,
1.737,0.3728,4.111,2.793,1.204,
1.239,0.3461,1.235,0.3933,1.987,
2.653,8.501,2.301,1.757,1.646,
5.68,2.508,0.4777,2.718,1.759,
1.362,0.63,3.016,1.632,5.634,
0.3583,1.388,0.6918,4.635,1.874,
4.194,2.134,1.384,2.0,4.244,
0.265,0.8989,1.945,1.862,4.98,
6.914,3.775,4.203),
cathl = c(
          NA,          NA,          NA,          NA,          NA,
          NA,          NA,0.9606,          NA,          NA,
          NA,          NA,          NA,          NA,          NA,
          NA,          NA,          NA,          NA,          NA,
          NA,          NA,          NA,          NA,          NA,
          NA,          NA,1.279,          NA,          NA,
          NA,          NA,          NA,          NA,          NA,
          NA,          NA,          NA,          NA,          NA,
          NA,          NA,          NA,          NA,          NA,
          NA,          NA,          NA,          NA,          NA,
          NA,          NA,          NA,1.416,          NA,
          NA,          NA,          NA,          NA,          NA,
          NA,0.3015,          NA,          NA,          NA,
          NA,          NA,          NA,          NA,          NA,
          NA,          NA,0.1978,          NA,          NA,
          NA,          NA,          NA,          NA,          NA,
          NA,          NA,          NA,          NA,          NA,
          NA,          NA,          NA,          NA,          NA,
          NA,          NA,          NA,          NA,          NA,
          NA,          NA,          NA,          NA,          NA,
          NA,          NA,          NA,          NA,          NA,
          NA,          NA,          NA,          NA,          NA,
          NA,          NA,          NA,          NA,          NA,
          NA,          NA,          NA,          NA,          NA,
          NA,          NA,          NA,          NA,          NA,
          NA,          NA,          NA,          NA,          NA,
          NA,          NA,          NA,          NA,          NA,
          NA,          NA,          NA,          NA,          NA,
          NA,          NA,          NA,          NA,          NA,
          NA,          NA,          NA,          NA,          NA,
          NA,          NA,          NA,          NA,          NA,
          NA,          NA,          NA),
```

```
cathlmore = c(
2.247,1.245,12.4,3.079,1.001,
0.6601,0.8775,1.854,1.487,0.9862,
0.5508,4.198,0.3974,2.213,0.6398,
0.7862,2.25,2.324,1.113,1.605,
0.3417,0.7307,0.8738,1.451,4.25,
3.209,3.475,2.447,1.778,0.3504,
1.278,0.8103,1.151,1.079,1.662,
1.712,2.078,1.821,1.38,1.346,
0.648,0.6947,0.923,0.3669,0.1254,
0.8052,0.4012,0.8312,0.6175,1.434,
1.054,1.389,0.3578,0.5946,0.7791,
0.5861,0.2175,0.7576,0.5149,0.7346,
0.09805,0.8423,0.5104,0.525,0.392,
1.216,0.2058,0.3366,0.2198,0.9608,
0.3711,0.327,1.23,0.1517,0.2919,
0.9025,1.257,0.4335,0.2678,0.8489,
0.3843,0.2885,0.3341,0.1444,0.1234,
0.7336,0.2835,0.3162,0.2496,0.328,
0.1776,0.2374,0.6711,0.5077,0.09841,
0.3869,0.3337,0.2936,0.3997,0.161,
0.3144,0.4061,0.4889,0.319,0.2282,
0.1774,0.7128,0.2068,0.1058,0.661,
0.4803,0.3155,0.09133,0.2758,0.308,
0.4858,0.7108,0.3687,0.4392,0.2629,
0.1437,0.1888,0.3641,0.2848,0.1742,
0.4504,0.4477,0.6919,0.4679,0.1728,
0.1545,0.181,0.2768,0.2134,0.3502,
0.5062,0.3454,0.5209,0.2562,0.2761,
0.3771,0.2291,1.421,0.1891,0.9043,
1.08,0.7077,0.2177,0.3405,1.194,
0.2937,0.0866,0.6069),
cpt1liver = c(
        NA,     NA,     NA,     NA,     NA,
        NA,     NA,     NA,     NA,     NA,
        NA,     NA,     NA,     NA,     NA,
        NA,     NA,     NA,     NA,     NA,
        NA,     NA,     NA,     NA,     NA,
        NA,     NA,     NA,     NA,     NA,
        NA,     NA,     NA,     NA,     NA,
        NA,     NA,     NA,     NA,     NA,
        NA,     NA,     NA,     NA,     NA,
        NA,     NA,     NA,     NA,     NA,
        NA,     NA,     NA,     NA,     NA,
        NA,     NA,     NA,     NA,     NA,
        NA,     NA,     NA,     NA,     NA,
        NA,     NA,     NA,1.301,     NA,
        NA,     NA,     NA,     NA,     NA,
        NA,     NA,     NA,     NA,     NA,
        NA,     NA,     NA,     NA,     NA,
        NA,     NA,     NA,     NA,     NA,
        NA,     NA,     NA,     NA,     NA,
        NA,     NA,     NA,     NA,     NA,
        NA,     NA,     NA,     NA,     NA,
0.07807,     NA,     NA,     NA,     NA,
        NA,     NA,     NA,     NA,     NA,
        NA,     NA,     NA,     NA,     NA,
        NA,     NA,     NA,     NA,     NA,
        NA,     NA,     NA,     NA,     NA,
        NA,     NA,     NA,     NA,     NA,
        NA,     NA,     NA,     NA,     NA,
        NA,     NA,     NA,     NA,     NA,
        NA,     NA,     NA,     NA,     NA,
        NA,     NA,     NA),
cpt1livermore = c(
2.45,0.5376,1.877,1.406,5.354,
3.116,1.76,1.026,3.295,4.69,
```

```
0.8916,6.127,1.73,1.874,3.855,
0.3075,2.085,5.489,2.578,3.299,
2.713,1.728,1.371,2.694,2.068,
2.135,4.108,3.05,2.014,1.875,
2.04,1.035,1.889,5.797,2.014,
1.645,3.659,2.342,1.851,2.281,
0.9387,0.7157,2.683,1.083,1.227,
0.6307,2.009,0.7853,1.71,1.643,
0.7674,1.829,1.784,1.726,1.272,
1.287,1.685,0.7701,1.763,1.086,
0.2811,0.6504,0.7742,0.3488,0.5319,
1.11,0.4093,0.7331,0.4523,0.5086,
0.4426,0.4097,0.5111,0.3882,0.3496,
2.132,0.4284,0.4796,0.7042,0.2756,
0.407,0.224,0.7151,0.5904,0.2921,
1.191,0.1206,0.4061,0.4251,0.6765,
0.1596,0.38,1.144,0.7264,0.2636,
0.5183,0.8161,0.6711,0.4942,0.3568,
0.3759,0.3492,0.4842,0.4743,0.2326,
0.6372,0.3564,0.3205,0.4433,0.3006,
0.08193,0.454,0.2697,0.3126,0.1592,
0.2134,0.3595,0.353,0.2409,0.5051,
0.1549,0.8434,0.5272,0.1342,0.356,
0.3201,0.1145,0.2549,0.2025,0.199,
0.2749,0.2537,0.5801,0.3067,0.3551,
0.2807,0.8103,0.5784,0.2349,0.9024,
0.5316,1.282,0.6575,0.6515,0.455,
0.2632,0.4885,0.1383,0.4512,0.1314,
0.2441,0.5905,0.1525),
d = c(
-0.08966,-1.811,-0.7629,0.3478,-0.3553,
1.795,-1.931,-23.57,-18.06,-18.77,
-22.97,-18.87,-32.45,-24.88),
e = c(
-0.02385,0.04771,1.461,-1.808,1.758,
0.6818,2.271,0.7007,-0.9067,-0.002554,
0.05133,-3.124,47.04,23.51),
f = c(
0.4894,0.05699,2.247,1.656,0.5941,
-0.8891,1.627,-2.837,-53.25,6.932,
-18.98,35.8,-24.39,26.5),
g = c(
0.1958,1.372,1.424,0.8955,0.5677,
0.2384,-1.803,30.17,-77.19,15.85,
-40.86,30.47,34.21,-21.16),
gamma = c(
1.039,1.255),
gdh = c(
      NA,      NA,      NA,1.301,      NA,
      NA,      NA,      NA,      NA,      NA,
      NA,      NA,      NA,      NA,      NA,
      NA,      NA,      NA,      NA,      NA,
      NA,      NA,      NA,0.8113,      NA,
      NA,      NA,      NA,      NA,      NA,
      NA,      NA,      NA,      NA,      NA,
      NA,      NA,      NA,      NA,      NA,
      NA,      NA,      NA,      NA,      NA,
      NA,      NA,      NA,      NA,      NA,
      NA,      NA,      NA,      NA,      NA,
      NA,      NA,      NA,      NA,      NA,
      NA,      NA,      NA,      NA,1.494,
      NA,      NA,      NA,      NA,      NA,
      NA,      NA,      NA,      NA,      NA,
      NA,      NA,      NA,      NA,      NA,
      NA,      NA,      NA,      NA,      NA,
      NA,      NA,      NA,      NA,      NA,
      NA,      NA,      NA,      NA,      NA,
```

```
	NA,	NA,	NA,	NA,	NA,
	NA,	NA,	NA,	NA,0.7273,
	NA,	NA,	NA,	NA,	NA,
	NA,	NA,	NA,	NA,	NA,
	NA,	NA,	NA,	NA,	NA,
	NA,	NA,	NA,	NA,	NA,
	NA,	NA,	NA,	NA,	NA,
	NA,	NA,	NA,	NA,	NA,
	NA,	NA,	NA,	NA,	NA,
	NA,	NA,	NA,	NA,	NA,
	NA,	NA,	NA,	NA,	NA,
	NA,	NA,	NA),
gdhmore = c(
0.933,1.007,0.9026,2.032,1.106,
0.9714,0.7762,1.161,0.4719,2.069,
0.4783,0.5147,1.667,0.6894,1.209,
1.027,0.6601,0.5657,1.101,0.9648,
0.6142,0.3706,0.7363,1.03,0.5628,
0.8346,0.3534,1.64,1.228,0.4533,
1.028,1.04,1.259,2.175,0.4646,
1.379,0.9678,0.9245,0.5791,0.4786,
1.118,2.469,1.095,0.5564,1.008,
1.463,0.6336,2.903,0.5627,1.113,
2.256,1.331,2.265,2.797,2.801,
3.704,1.373,2.72,1.585,1.639,
0.5831,1.766,0.9254,1.515,0.767,
1.696,1.529,2.023,0.93,1.329,
1.921,0.9914,1.767,0.9688,2.305,
1.012,1.429,2.366,1.319,0.8865,
0.7497,0.4089,1.237,0.9454,0.9053,
0.8531,0.9471,0.6765,1.982,1.203,
0.8554,0.8731,0.6744,0.6413,1.409,
0.2677,0.4368,0.6422,1.468,0.9705,
2.781,0.8403,0.8193,0.8714,0.4914,
1.346,0.8218,0.7355,0.7104,0.7429,
1.536,0.9454,0.5528,1.202,0.6121,
0.7578,0.631,0.7361,0.1467,0.8808,
0.4526,0.5689,0.8925,1.625,0.8535,
0.5805,0.4831,1.099,0.3277,1.314,
1.056,2.096,1.25,1.544,0.5934,
2.148,0.7171,0.9874,1.536,2.049,
0.8046,0.9041,1.473,0.5952,1.409,
0.5126,1.647,1.753,0.5598,0.8039,
1.501,1.228,2.385),
h = c(
-0.7384,-4.949,2.754,-11.71,-27.94,
-41.62,-2.271,15.39,-23.08,0.3507,
-9.73,18.8,32.7,25.31),
lc3b = c(
	NA,	NA,	NA,	NA,	NA,
	NA,	NA,	NA,	NA,	NA,
	NA,	NA,	NA,	NA,	NA,
	NA,	NA,	NA,0.6951,	NA,
	NA,	NA,	NA,	NA,	NA,
	NA,	NA,	NA,	NA,	NA,
	NA,	NA,	NA,	NA,	NA,
	NA,	NA,	NA,1.043,	NA,
	NA,	NA,	NA,	NA,	NA,
	NA,	NA,	NA,	NA,	NA,
	NA,	NA,	NA,	NA,	NA,
	NA,1.257,	NA,	NA,	NA,
	NA,	NA,	NA,	NA,	NA,
	NA,	NA,	NA,	NA,	NA,
	NA,	NA,	NA,	NA,	NA,
	NA,	NA,0.5739,	NA,	NA,
	NA,	NA,	NA,	NA,	NA,
	NA,	NA,	NA,	NA,	NA,
```

```
        NA,        NA,        NA,        NA,        NA,
        NA,        NA,        NA,        NA,        NA,
        NA,        NA,        NA,        NA,        NA,
        NA,        NA,        NA,        NA,        NA,
        NA,        NA,        NA,        NA,        NA,
        NA,        NA,        NA,        NA,        NA,
        NA,        NA,        NA,        NA,        NA,
        NA,        NA,        NA,        NA,        NA,
        NA,        NA,        NA,        NA,        NA,
        NA,        NA,        NA,        NA,        NA,
        NA,        NA,        NA,        NA,        NA,
        NA,        NA,        NA,        NA,        NA,
        NA,        NA,        NA),
lc3bmore = c(
0.788,1.898,0.6423,0.7776,1.168,
1.188,0.8602,1.044,0.7296,1.204,
1.417,1.296,1.14,1.073,1.356,
1.95,1.475,1.146,0.99,0.6932,
0.9223,0.4387,1.011,1.279,1.606,
0.7246,0.6819,1.558,0.6173,0.7227,
0.8418,1.193,0.7962,0.8322,0.8531,
1.124,0.9531,0.8301,0.6367,1.176,
1.498,1.411,1.079,0.6289,1.552,
1.101,1.521,1.411,0.2388,1.425,
0.8294,1.236,0.7601,2.922,0.9539,
1.217,1.122,0.6244,1.697,1.198,
0.895,0.4252,1.149,0.2388,0.3601,
0.5111,1.071,1.23,1.039,0.3859,
1.249,0.7855,0.6109,0.4828,0.5454,
0.6899,1.052,0.4935,0.3186,0.3014,
0.6657,0.8075,0.3774,1.172,1.333,
0.6588,0.9036,0.549,0.5265,0.2683,
0.6396,0.4647,0.7426,0.8954,0.69,
0.3189,0.3318,0.7506,0.7563,0.9254,
0.7771,0.3549,0.5435,0.3842,0.3511,
0.4754,0.6699,0.621,0.7214,0.3781,
0.6696,0.3651,0.421,0.5032,0.6606,
0.1409,1.18,0.7761,0.5721,0.442,
0.976,0.3848,0.8429,0.7662,0.8563,
0.6095,0.4032,0.2226,0.6649,0.7012,
0.3926,0.7816,0.5665,0.4996,0.5193,
1.012,0.672,0.5917,0.454,0.5081,
0.8833,0.6107,0.7684,0.315,1.23,
0.9962,0.4831,0.4467,0.4322,0.5399,
0.4469,0.5101,0.6271),
m = c(
-0.02915,0.1554,-0.6401,-0.8154,0.8186,
-0.5267,0.3542,1.051,2.084,-2.536,
-25.79,28.08,48.99,3.741),
ma12p = c(
1.921,1.909,1.108,1.017,1.235,
2.333,1.32,0.5233,2.251,0.6818,
1.076,0.3008,0.06335,1.884,0.5186,
0.294,0.1557,0.2647,0.4432,0.3299,
0.5722,0.5656,0.3117,0.1792,0.2293,
0.2095,0.2706,0.6093,0.3885,0.4865,
0.1707,0.3322,0.4364,0.2281,0.3404,
0.5539,0.07673,0.2028,0.1747,0.1992,
0.3099,0.2375,0.595,0.3824,0.4211,
0.5779,0.6882,0.4765,0.659,0.4281,
0.5344,0.5966,0.7647,0.7356,1.178,
0.268,0.3344,0.8454,1.558,0.4703),
ma9p = c(
2.303,3.604,1.244,0.3904,0.1552,
0.3985,0.5718,0.9229,0.5692,1.676,
0.7257,0.3763,1.46,0.452,0.5512,
0.6903,0.5238,0.6597,0.4074,0.5665,
```

```
0.7075,0.2819,0.4002,0.281,0.4597,
0.4974,0.2418,1.053,0.9533,0.467,
0.1924,0.3098,0.3842,0.1819,0.2493,
0.3052,0.6787,0.2817,0.3708,0.3188,
0.1798,0.2957,0.737,0.3133,0.7585,
0.07592,0.1355,0.3915,0.6208,0.7423,
0.3685,1.032,2.203,1.61,0.4308,
1.277,0.3349,0.6343,0.1945,0.642),
mb12p = c(
4.103,4.25,0.6287,0.9253,0.5,
0.2715,0.6184,0.591,0.4551,0.255,
0.4087,0.6311,0.6576,0.7607,0.545,
0.6171,0.5589,0.4149,0.4573,1.458,
0.8093,0.8601,1.189,0.751,0.8129,
0.7658,0.9814,0.5516,1.357,0.985,
0.942,0.9686,0.7866,0.6843,0.9059,
1.15,0.7135,0.8441,1.018,0.5768,
0.6753,1.233,1.083,0.9794,0.6098,
1.831,1.286,2.226,2.077,1.142,
2.976,3.445,1.781,3.429,1.745,
3.05,2.572,3.277,4.543,1.022),
mb9p = c(
2.112,1.954,0.6694,0.419,0.8159,
0.7033,1.209,0.8211,0.7131,0.5171,
1.146,0.5155,0.4323,0.3522,0.4636,
0.2898,0.2839,0.3939,0.2824,0.4627,
0.3396,0.36,0.4655,0.3471,0.7527,
0.451,0.3477,0.587,1.311,0.6462,
0.57,1.231,0.5335,0.7901,0.6447,
1.282,0.7972,1.988,2.234,1.446,
2.672,1.164,2.545,2.689,1.661,
1.929,1.316,1.209,1.141,0.7131,
1.19,2.409,1.061,2.615,1.623,
0.9483,1.112,1.59,2.68,5.438),
mc12p = c(
0.9038,1.923,1.22,1.624,1.815,
1.935,2.693,1.399,1.544,1.631,
2.307,0.8226,2.007,0.6614,0.657,
0.9744,0.7004,0.5836,0.2458,0.6442,
0.5802,0.8023,0.5854,0.3146,0.6737,
0.7852,0.412,0.8537,0.5095,0.5108,
0.6974,0.431,0.5366,0.3786,0.9655,
0.215,0.9019,1.555,0.2585,0.4317,
0.3892,0.5014,0.3804,0.5032,0.2789,
0.3798,0.7942,0.2937,0.8801,0.5757,
0.3342,0.3945,2.022,0.4404,0.4724,
0.9456,0.2569,0.4752,1.131,0.7327),
mc9p = c(
1.004,1.164,1.104,0.772,1.216,
1.098,1.179,1.421,2.007,0.8796,
1.399,2.664,1.476,0.6259,1.205,
0.724,1.374,0.9998,0.3627,0.5635,
0.5079,0.901,0.615,0.464,0.6725,
0.4334,0.3365,1.055,0.7991,0.2253,
0.3805,0.4718,0.8322,0.4791,0.2954,
0.3685,0.9554,0.3933,0.5803,0.2488,
0.3467,0.3971,0.2197,0.5074,0.3825,
0.6729,0.3702,0.3102,0.4779,1.15,
0.3588,0.5227,0.6863,1.307,0.9385,
0.9156,0.337,0.4326,0.542,0.3998),
md12p = c(
0.2874,0.43,0.7966,1.101,2.515,
1.592,1.789,2.639,2.999,2.517,
2.548,1.319,0.9228,1.169,0.4986,
1.376,1.21,0.9363,0.5853,0.9077,
0.6848,0.496,0.8073,0.453,0.8337,
1.726,1.238,0.4629,2.642,2.1,
```

```
2.066,1.228,0.917,2.114,1.601,
0.6132,1.517,1.267,2.459,0.9185,
1.332,0.612,0.3934,0.8847,0.6859,
0.6177,1.029,0.2789,0.3965,0.7937,
0.9404,0.3481,0.4211,0.609,0.9348,
0.4833,0.6776,0.6891,0.5566,0.3464),
md9p = c(
0.2069,0.391,1.581,0.7748,1.685,
2.06,3.04,1.97,1.992,1.161,
1.242,0.9942,2.793,0.6296,0.7251,
1.46,0.7563,0.6077,0.4884,0.4799,
0.4785,1.776,0.2765,0.5521,0.5956,
1.101,1.077,1.975,1.192,1.307,
0.9936,0.6087,1.655,1.585,1.157,
1.017,0.7669,0.5061,1.819,1.402,
1.959,2.143,0.8829,1.084,1.158,
0.4759,1.481,1.24,0.7813,0.4759,
0.2297,1.303,0.8785,1.039,0.5111,
0.3857,0.3206,0.4929,0.7502,0.2986),
me12p = c(
0.9651,0.1699,0.1691,0.2279,0.8044,
0.3018,0.4897,0.468,0.5585,1.039,
0.7714,1.002,0.8151,3.511,1.104,
1.059,0.8216,1.549,1.61,1.784,
1.805,0.5155,0.9088,1.337,2.02,
0.9837,1.966,1.015,1.272,0.78,
1.203,0.854,0.5439,2.233,1.51,
3.456,2.028,0.8791,1.521,1.024,
0.9668,1.145,1.539,1.299,0.7148,
1.016,1.214,0.4754,1.355,1.714,
1.229,1.543,0.9279,0.7192,1.973,
1.037,1.62,2.421,0.2943,0.7187),
me9p = c(
0.1335,0.2085,0.4192,0.5841,1.175,
0.5893,0.5337,0.4665,0.6757,1.01,
2.541,0.6644,0.5389,1.092,1.365,
1.175,0.5236,1.7,1.011,1.019,
0.3081,0.8616,0.9897,1.467,1.259,
1.127,0.4295,0.5017,1.185,1.765,
2.381,1.518,1.406,1.473,1.38,
2.163,1.83,1.682,3.01,1.401,
1.36,1.71,2.041,1.358,0.5534,
1.074,0.5789,2.136,1.144,1.506,
0.6207,0.8137,0.6908,0.5068,1.002,
0.6725,0.9787,1.527,2.391,2.774),
mf12p = c(
0.1984,0.1268,0.6473,1.309,6.359,
3.092,6.812,7.813,7.179,3.065,
3.987,1.296,1.686,2.776,0.9185,
1.35,2.118,1.651,0.7553,0.8176,
1.458,1.343,0.8004,0.7561,1.926,
0.8344,1.299,0.7594,1.758,2.092,
1.814,1.186,3.066,1.741,1.565,
1.952,5.738,2.624,0.7678,0.8304,
1.527,2.231,2.397,0.6287,8.073,
0.5691,1.239,1.114,3.027,2.241,
5.091,1.802,3.37,5.127,2.68,
3.21,5.764,4.931,9.93,3.142),
mf9p = c(
0.1102,0.8251,3.317,1.132,4.174,
5.789,2.837,1.235,2.113,3.578,
2.599,2.8,1.302,1.108,0.5763,
0.7277,0.4692,0.4371,0.6567,2.01,
0.5324,4.543,0.9793,0.9604,0.8481,
0.3976,1.419,0.9923,1.267,1.109,
8.615,3.091,2.524,3.934,0.3362,
0.9263,0.7677,0.3379,1.046,3.126,
```

```
0.4401,2.098,3.391,1.93,3.215,
1.634,5.956,2.144,14.49,3.754,
2.6,0.8275,0.946,5.692,0.9647,
2.536,0.7519,5.34,0.6533,4.789),
mg12p = c(
0.6861,0.9836,0.8159,1.023,1.021,
1.858,0.3726,4.124,0.6534,1.66,
0.321,0.9387,0.2991,1.063,0.8037,
0.9995,0.6311,0.8533,2.542,4.219,
1.003,1.016,1.791,1.398,0.3952,
2.167,2.057,1.653,6.211,0.8808,
0.3979,0.9877,0.6991,0.906,1.019,
1.52,2.666,0.5864,1.159,1.708,
0.2324,2.17,0.2203,0.366,0.4443,
0.6954,0.3232,0.6019,1.009,2.289,
1.641,0.216,3.156,1.245,1.425,
1.741,10.18,2.878,3.468,1.035),
mg9p = c(
1.3,1.685,0.9199,0.3837,1.752,
1.015,1.376,1.51,0.5418,1.313,
1.85,0.6576,1.896,1.126,1.915,
0.8072,0.6042,0.3237,0.6794,0.3257,
0.8756,0.3319,0.66,1.421,0.2022,
0.7058,0.3408,0.3433,0.6338,0.4337,
1.338,0.484,0.47,0.8301,0.8114,
3.158,0.5291,0.7603,0.3723,0.3848,
1.384,0.3509,1.023,2.01,0.3077,
0.5595,1.287,1.675,0.7212,1.089,
0.6354,1.252,1.113,4.29,3.776,
11.85,3.446,2.264,4.253,1.624),
mh12p = c(
1.988,1.893,1.326,1.865,1.684,
5.677,1.112,1.434,1.98,1.716,
0.5344,0.4926,0.7169,0.7235,0.3818,
2.485,1.032,0.3183,0.407,0.9169,
1.838,0.3447,0.1056,0.7701,2.08,
0.5733,0.3914,1.211,0.3116,0.7293,
0.2227,0.1815,0.4311,0.4499,0.3186,
0.5326,0.3276,0.7696,0.5196,0.1636,
0.2328,0.1692,0.09905,0.2151,0.2351,
0.1933,0.2798,0.6427,0.1597,0.2016,
0.2093,0.1752,0.4214,0.1964,0.5824,
0.2607,0.4366,0.3681,0.2085,0.3215),
mh9p = c(
6.799,1.138,1.382,4.842,0.8795,
1.307,4.06,1.516,1.144,0.7407,
0.6709,0.3165,0.9116,0.9808,0.7389,
0.6805,0.9196,1.799,0.3688,0.5147,
0.1495,0.4095,0.1563,1.117,0.2863,
0.2562,0.4398,0.4368,0.3671,0.3063,
0.2246,0.07982,0.169,0.5124,0.2866,
0.4673,0.1729,0.2442,0.164,0.2476,
0.405,0.2024,0.1373,0.1566,0.0693,
0.2431,0.1726,0.1291,0.2876,0.4723,
0.1944,0.376,0.09844,0.5188,0.39,
0.2587,0.1616,0.8061,0.246,0.08188),
mm12p = c(
0.297,0.3467,1.157,0.4249,0.8441,
1.133,0.6032,1.79,1.261,1.741,
5.519,7.505,4.252,4.856,1.406,
1.2,1.174,2.798,1.434,1.458,
1.055,0.879,1.016,0.8246,1.161,
1.535,0.3635,0.5984,0.4731,0.9196,
1.096,0.6416,0.4654,0.8826,0.6239,
0.5293,0.3293,0.6136,0.3927,0.7822,
0.6065,0.9782,0.6593,1.187,0.5706,
0.7525,1.448,1.698,1.182,2.233,
```

```
1.464,1.078,0.8038,0.3961,0.8486,
2.004,2.222,1.313,1.141,0.6136),
mm9p = c(
0.7621,2.003,0.776,0.2727,1.719,
0.6776,0.6991,2.001,0.9213,0.4458,
1.522,1.959,0.6063,1.324,3.168,
1.036,1.382,0.6393,0.8669,1.187,
0.9146,1.541,1.215,1.19,1.56,
1.037,0.4981,1.296,0.6502,0.9668,
1.104,0.4645,2.866,0.7832,1.45,
1.083,0.8597,0.8981,0.8236,1.918,
1.29,1.592,1.619,1.091,0.9994,
0.7738,1.745,1.268,2.745,0.3583,
1.053,0.8469,0.5467,1.513,0.6973,
0.854,1.573,0.8315,0.6397,1.363),
mn12p = c(
1.855,5.16,5.039,3.499,5.035,
4.311,3.985,0.7037,1.812,4.327,
1.887,1.134,2.292,1.56,2.1,
0.6805,0.676,0.8144,1.156,0.5405,
0.4671,0.331,0.5813,0.5637,2.522,
0.6477,1.244,0.3371,0.2666,0.1563,
0.5225,0.6621,1.17,0.591,0.3157,
0.4261,0.4444,0.3906,0.2353,0.494,
0.411,0.2031,2.305,0.2064,0.2016,
0.1036,0.1845,0.3647,0.4004,0.1581,
0.1795,0.2319,1.206,0.2044,0.1306,
0.2835,0.1447,0.1512,0.3248,0.2071),
mn9p = c(
1.962,5.685,1.554,4.049,5.402,
3.173,1.422,4.797,3.059,2.136,
0.8595,1.365,1.418,1.324,1.713,
2.458,1.869,0.8532,0.3144,0.8946,
1.167,1.314,2.31,0.6231,0.3909,
0.1148,0.2854,0.6454,0.2783,0.8877,
0.3188,0.1492,0.7147,0.2517,0.5344,
0.2393,0.4432,0.2417,0.6764,0.2701,
0.3219,0.1604,0.1693,0.3711,0.2976,
0.1419,0.1288,0.3173,0.1968,0.1562,
0.2294,0.1782,0.1299,0.2084,0.5758,
0.4799,0.3532,0.7992,0.09478,0.3828),
n = c(
-0.5152,0.0525,-6.445,2.473,-0.7613,
0.1034,-36.67,6.984,-11.53,-42.65,
-12.68,16.27,83.04,-17.05),
p = c(
0.1,0.2,0.2,0.2,0.1,0.2),
q = c(
0.1,0.2,0.2,0.2,0.1,0.2),
random = c(
0.8002,0.5392,0.2805,3.305,2.236,
0.5492,1.247,0.7801,0.9591,0.5122,
1.676,0.9416,0.6356,0.9443,1.59,
0.45,1.414,0.25,0.6069,0.7867,
1.311,1.831,0.6946,1.082,0.5576,
1.202,0.8592,0.6387,1.367,1.037,
0.7952,1.671,1.104,0.5877,0.9293,
0.342,2.121,1.184,0.7083,0.2032,
0.9607,0.9625,0.9035,0.6374,0.7184,
0.9366,1.079,0.6646,0.7866,0.9018,
1.005,1.535,1.457,0.9293,0.7381,
1.312,0.8786,0.602,1.078,1.077,
0.3726,0.7745,0.6755,0.5714,0.6239,
0.5692,0.7728,1.567,0.6261,0.9024,
0.4442,0.8383,0.7203,0.9682,1.119,
0.4192,0.5516,1.474,0.5854,0.3247,
0.8302,0.714,1.332,0.3861,0.8991,
```

```
0.7579,2.239,1.054,0.878,1.04,
0.8754,0.8389,0.4643,1.064,1.024,
0.5018,1.372,0.5522,0.758,1.403,
0.6183,1.073,0.6397,0.5297,1.017,
0.3868,0.5036,5.135,0.3981,1.564,
0.8446,0.3658,1.339,0.6709,0.4621,
1.015,0.9542,0.5714,0.4857,0.7304,
0.6567,3.584,0.6497,0.5149,1.147,
1.073,0.9046,2.201,0.5846,0.6989,
0.8548,0.4996,1.313,2.356,0.6744,
0.5134,0.3878,1.674,0.6467,0.4214,
2.66,0.5765,0.8963,1.095,0.5311,
1.359,0.3493,0.603,1.035,0.8075,
1.001,0.6808,1.282),
u1 = c(
2.765,3.165,-10.97,-80.48,39.11,
-5.013,-59.08,27.49,24.13,-22.72,
30.32,-60.34,39.51,-1.724,84.33,
0.2884,-12.5,-17.2,-10.82,-57.12,
51.99,24.73,-23.48,-59.32),
u2 = c(
1.755,2.32,5.701,-7.59,17.32,
-75.66,30.2,7.99,-23.33,-19.96,
2.214,-0.05528,-4.6,-58.34,-30.57,
31.37,-14.44,10.35,-40.96,-3.496,
-10.51,55.82,-7.585,-66.62),
ulk1 = c(
      NA,      NA,      NA,      NA,      NA,
      NA,      NA,      NA,      NA,      NA,
      NA,      NA,      NA,      NA,      NA,
      NA,      NA,      NA,0.5285,      NA,
      NA,      NA,      NA,      NA,      NA,
      NA,      NA,      NA,      NA,      NA,
      NA,      NA,      NA,      NA,      NA,
      NA,      NA,      NA,0.7154,      NA,
      NA,      NA,      NA,      NA,      NA,
      NA,      NA,      NA,      NA,      NA,
      NA,0.486,      NA,      NA,      NA,
      NA,1.196,      NA,      NA,      NA,
      NA,0.123,      NA,      NA,      NA,
0.2558,      NA,      NA,      NA,      NA,
      NA,      NA,      NA,      NA,      NA,
      NA,      NA,      NA,      NA,      NA,
      NA,      NA,      NA,      NA,      NA,
      NA,      NA,      NA,      NA,      NA,
      NA,      NA,      NA,      NA,      NA,
      NA,      NA,      NA,      NA,      NA,
      NA,      NA,      NA,      NA,      NA,
      NA,      NA,      NA,      NA,      NA,
      NA,      NA,      NA,      NA,      NA,
      NA,      NA,      NA,      NA,      NA,
      NA,      NA,      NA,      NA,      NA,
      NA,      NA,      NA,      NA,      NA,
      NA,      NA,      NA,      NA,      NA,
      NA,      NA,      NA,      NA,      NA,
      NA,      NA,      NA,      NA,      NA,
      NA,      NA,      NA,      NA,      NA,
      NA,      NA,      NA),
ulk1more = c(
1.149,0.5146,2.217,1.447,2.029,
1.056,0.8872,0.7691,1.273,0.4611,
0.8401,0.6825,1.043,0.2909,0.6795,
0.3115,1.515,1.384,0.487,0.661,
0.6088,0.3431,1.556,0.6434,3.047,
1.41,1.501,0.7479,0.6605,0.401,
1.194,1.335,0.7698,1.379,0.6345,
2.668,1.28,1.134,1.958,1.218,
```

```
0.299,0.4799,0.156,0.2652,0.9939,
1.45,0.1964,0.4722,0.8589,0.3457,
2.496,1.037,0.3967,1.107,0.5891,
0.3372,1.714,0.36,0.8665,0.1424,
0.1107,0.3111,0.1075,0.315,0.3978,
0.1464,0.4689,0.4068,0.285,0.389,
0.1667,0.2574,0.17,0.1925,0.2962,
0.251,0.293,0.8641,0.668,0.6221,
0.4059,0.242,0.3355,1.625,0.1838,
0.5324,0.5117,0.1688,0.3306,0.4641,
0.1583,0.5105,0.155,0.3943,0.2615,
0.1645,0.2564,0.3421,0.2517,0.2788,
0.3151,0.4733,0.1862,0.3406,0.418,
0.3687,0.1931,0.2098,0.3164,0.8118,
0.1384,0.4286,0.4673,0.1755,0.5734,
0.2273,0.8578,0.511,0.1867,0.2799,
1.125,0.8593,0.6325,1.643,0.581,
0.2344,0.9075,0.4269,0.4502,1.215,
1.155,0.7735,0.2204,1.459,0.6585,
0.8323,0.3689,0.6498,0.6694,0.4682,
0.3982,0.3134,0.396,0.3373,0.5745,
0.7358,0.9554,0.5255,0.4885,0.446,
0.5008,0.4758,0.8268),
v1 = c(
0.07201,0.0396,1.194,68.93,26.52,
-27.59,-19.38,52.45,27.46,-13.85,
-24.34,57.55,-18.92,17.6,-2.097,
-4.34,9.68,20.01,-4.847,-8.076,
-30.08,-5.526,65.57,16.56),
v2 = c(
0.08909,0.07831,-14.71,-49.12,34.2,
-13.36,56.2,37.64,4.463,-19.72,
-26.03,74.53,-4.815,-20.41,-29.25,
-7.65,-2.695,0.3833,50.13,-14.92,
22.7,49.92,-6.498,6.614),
vara = 0.3916,
varb = 0.1312,
varc = 0.1736,
vard = 0.2546,
vare = 0.2141,
varf = 0.6062,
varg = 0.5902,
varh = 0.4514,
varm = 0.2165,
varn = 0.3222,
w12p = c(
3.353,3.266,6.91,10.05,12.57,
17.56,13.22,15.63,13.85,25.05,
19.41,26.71,23.04,26.21,25.98,
23.85,29.17,21.79,41.08,30.9,
20.06,29.35,36.15,33.3,36.28,
24.35,35.95,31.16,46.8,43.42,
32.29,53.68,38.51,71.5,46.24,
59.45,68.65,67.48,78.16,41.37,
65.54,65.84,69.45,65.53,77.78,
67.23,77.57,85.9,66.52,78.5,
72.75,56.07,86.8,80.94,121.7,
85.13,128.5,79.44,65.03,84.92),
w9p = c(
2.282,5.057,4.654,0.641,4.617,
5.0,13.65,2.604,6.216,7.667,
6.398,14.59,13.54,18.59,6.281,
16.2,11.2,7.567,13.28,14.78,
34.14,27.59,18.51,11.94,30.22,
16.43,22.36,34.53,25.72,36.36,
45.9,29.63,33.83,37.83,28.48,
23.31,22.66,22.57,25.52,62.24,
```

51.26,59.88,70.42,42.97,40.13,
41.0,105.0,27.62,19.98,51.47,
31.12,48.47,58.45,90.16,68.78,
101.8,100.2,87.24,65.23,88.29))
